# Chimeric DCL1-Partnering Proteins Provide Insights into the MicroRNA Pathway

**DOI:** 10.3389/fpls.2015.01201

**Published:** 2016-01-06

**Authors:** Rodrigo S. Reis, Andrew L. Eamens, Thomas H. Roberts, Peter M. Waterhouse

**Affiliations:** ^1^School of Biological Sciences, University of Sydney, SydneyNSW, Australia; ^2^Department of Plant and Food Sciences, Faculty of Agriculture and Environment, University of Sydney, SydneyNSW, Australia; ^3^School of Environmental and Life Sciences, University of Newcastle, CallaghanNSW, Australia; ^4^Centre for Tropical Crops and Biocommodities, Queensland University of Technology, BrisbaneQLD, Australia

**Keywords:** DRB1, HYL1, DRB2, miRNA, dsRNA binding domain, chimera

## Abstract

In *Arabidopsis thaliana*, efficient microRNA (miRNA) production requires DICER-LIKE1 (DCL1) with the assistance of a partnering protein, DOUBLE-STRANDED RNA BINDING1 (DRB1) or DRB2. The presence of either of these DRB proteins is crucial to determine the mode of action of a miRNA; i.e., cleavage or translation inhibition. Here we studied the structural determinants for the role of DRB1 and DRB2 in the miRNA pathway. We developed a series of chimeric vectors encoding different functional domains of DRB1 and DRB2, and expressed these in the *drb1* mutant background in *Arabidopsis* under the control of the native DRB1 promoter. Complementation of the *drb1* developmental phenotype was used to assess the biological role that each functional domain of DRB1 and DRB2 mediates in the miRNA-guided transcript cleavage pathway. The DRB1 amino acid sequence differs considerably to that of DRB2, and analysis of *drb1* transgenic lines revealed that the first dsRNA-binding domains of DRB1 and DRB2 are functionally similar; in contrast, the dsRBD2 of DRB1 and DRB2 appear functionally distinct. Our bioinformatic analysis further suggests that the C-terminal domain of DRB2 mediates a functional role in the miRNA pathway, whereas its counterpart in DRB1 is known to be dispensable. Our results provide evidence for the differences between DRB1 and DRB2 proteins *in vivo*, which may be essential for the selection of the miRNA regulatory mechanisms, and suggest that these features are conserved among land plants.

## Introduction

DOUBLE-STRANDED RNA BINDING1 (DRB1) is a well characterized partnering protein of DCL1 and is required for accurate and efficient processing of miRNA/miRNA^∗^ duplexes from their respective precursor transcripts (Kurihara et al., 2006; Dong et al., 2008). DRB1 also mediates the preferential selection of miRNA guide strands over the corresponding duplex strand (the miRNA^∗^ passenger strand) for loading into the ARGONAUTE1 (AGO1)-catalyzed RNA-induced silencing complex (RISC; Eamens et al., 2009). DRB2 is also a DCL1 partnering protein (Eamens et al., 2012). We have recently shown that DRB1 is required only for miRNA-guided transcript cleavage, whereas DRB2 represses *DRB1* transcription and is required for the translational inhibition pathway (Reis et al., 2015). Thus, although DCL1 is the ribonuclease III that excises miRNA/miRNA^∗^ duplexes from precursor transcripts, its DRB partnering protein defines the miRNA mode of action.

In *Arabidopsis*, genes encoding three additional DRB proteins—DRB3, DRB4, and DRB5—are present and each DRB is characterized by two amino-terminal dsRNA-binding domains (dsRBDs; Curtin et al., 2008). dsRBD-containing proteins have been identified in most eukaryotes, are typically 70 amino acid residues in length, fold into an αβββα structure, mediate dsRNA recognition and binding, and can also bridge protein-protein interactions (Chang and Ramos, 2005). In a canonical dsRNA binding domain, three regions of contact with the dsRNA molecule can be distinguished: regions 1 and 2 bind the dsRNA minor groove, while region three binds the major groove (Gleghorn and Maquat, 2014). The regions are characterized by positive electrostatic surface, with region two featuring a GPxH motif.

The structures of both dsRBD domains of DRB1, termed dsRBD1 and dsRBD2, respectively, have been determined (Yang et al., 2010). These revealed that dsRBD1 adopts a canonical dsRBD structure; i.e., it has the structural features found in most dsRNA binding domains. Interestingly, dsRBD2 is structurally distinct and its predicted low affinity for the dsRNA substrates of DRB1 has been explained by deviations from the canonical structure in region 2 (the loop that recognizes the dsRNA minor groove), and to a lesser extent in the α-helices that recognize both the minor and major dsRNA grooves (Yang et al., 2010). The same authors also showed that DRB1 binds 21-nucleotide (nt) dsRNA as a homodimer, probably mediated by its dsRBD2. More recently, DRB1 homodimerization has been further characterized, with the role of dsRBD2 shown to be crucial to ensure the position of DCL1-catalyzed pri-miRNA cleavage (Yang et al., 2014). Surprisingly, disruption of DRB1 homodimerization did not impair DRB1 interaction with DCL1, or the pri-miRNA binding affinity of DRB1.

Engineered chimeric genes coding for different combinations of dsRBDs have been widely used to study their functions in humans and plants. In humans, a single dicer protein processes both pre-miRNAs and pre-siRNAs in association with DRB proteins, namely protein activator of PKR (PACT) (Lee et al., 2006) and *trans*-activation response RNA-binding protein (TRBP; Chendrimada et al., 2005; Haase et al., 2005), respectively. Animal miRNAs primarily guide translation inhibition, while short interfering RNAs (siRNAs) guide transcript cleavage. Lee et al. (2013) showed that in humans this dichotomy is partially aided by Dicer association with either PACT or TRBP. PACT was found to inhibit the processing of pre-siRNA substrates by Dicer, and experiments with chimeric versions of PACT and TRBP demonstrated that the two N-terminal RNA-binding domains of each protein confer their differential function. Chimeric versions of DRB proteins have also been used to unravel the structural components of *Caenorhabditis elegans* RDE-4 required to bind dsRNA, interact with dicer DCR-1 and activate DCR-1 (Parker et al., 2008). Interestingly, a chimeric DRB protein containing the canonical TRBP2 dsRBD2 fused to the non-canonical DRB1 dsRBD2 compensated for DRB1 dsRBD1 by rescuing the *drb1* severe phenotype (Yang et al., 2010), providing evidence that certain dsRNA binding domains are primarily involved in the recognition and binding of dsRNA; e.g., DRB1 dsRBD1.

In this study, we demonstrate that the dsRBD1 domain of DRB1 is functionally similar to the corresponding domain in DRB2, whereas their dsRBD2 domains are functionally distinct. We also show that, while the C-terminal of DRB1 appears functionally redundant, its counterpart in DRB2 is functional. These results provide insights into the structural determinants of DRB1 and DRB2 activity *in vivo*, and into the miRNA-guided transcript cleavage pathway.

## Materials and Methods

### Plant Lines and Growth Conditions

The *drb1* T-DNA knockout insertion has been described previously (Curtin et al., 2008; Eamens et al., 2012). Plant lines were cultivated under standard growth conditions of 16 h light/8 h dark at a constant temperature of 24°C. Prior to soil transfer, all *Arabidopsis* lines were germinated on Murashige and Skoog (MS) agar media containing 1% sucrose for PCR-based genotyping to confirm genetic background. DNA oligonucleotides used as primers for PCR-based genotyping are listed in Supplementary Material.

### Germination Under Abscisic Acid (ABA) Treatment

*Arabidopsis* Col-0 wild-type, mutant and transgenic line seeds were placed on filter paper saturated with either water (control) or 0.5 μM abscisic acid (ABA), incubated at 4°C for 48 h, and then transferred to growth cabinets for germination under standard growth conditions.

### Protein Sequence Alignment

Putative *Arabidopsis thaliana* DRB1 and DRB2 ortholog protein sequences were obtained using the Phytozome database (Goodstein et al., 2012) and the basic local alignment search tool (BLAST; Altschul et al., 1990). Sequence alignments were performed using the default parameters of Clustal W (Larkin et al., 2007). The evolutionary tree time scale was based on a previous report (Clarke et al., 2011). The complete list of ortholog proteins is presented in Supplementary Material.

### Prediction of dsRNA Binding Domain Structure

Secondary structure predictions for dsRNA binding domains of DRB2, DRB3, DRB4 and DRB5 were performed using the default parameters of the I-TASSER online server (Roy et al., 2010).

### Construction of Expression Vectors and Plant Transformation

The construction of the expression vectors used to transform *drb1* mutants was performed using standard cloning techniques using Gateway^®^ cloning (Invitrogen) and synthesized DNA sequences. The binary vector used to transform the plants was a Gateway vector, pKCTAP, obtained from Plant Systems Biology (VIB, Belgium; http://gateway.psb.ugent.be/), which has been previously described (Van Leene et al., 2007). The selectable marker cassette from pORE-O1 (Coutu et al., 2007), containing a *Pat* gene driven by *P_*HPL*_* (*A. thaliana* hydroperoxide lyase promoter), was amplified using oligos that contained overhanging restriction sites for *Rsr*II at both 5′ and 3′ ends. pKCTAP was digested with *Rsr*II and the *Pat* gene cassette was sticky-end ligated.

To prepare the gene constructs (chimeras) to be inserted into the modified pKCTAP, a series of ∼500 nt DNA sequences, termed gBlocks^®^, were designed in-house and synthesized by Integrated DNA Technologies (IDT). Each gBlock contained sequences coding the dsRNA binding domain of DRB1 and/or DRB2 as listed and described in Supplementary Material. The gBlocks were designed to contain 5 *Hind*III and *Mfe*I and a 3 *Nhe*I restriction site, which were subjected to restriction enzymatic digestion, followed by ligation, to aid their insertion into a vector containing either the *DRB1* (*Mfe*I and *Nhe*I) or *DRB2* (*Hind*III and *Nhe*I) sequence. The obtained constructs, as well as the dsRNA binding domains of *DRB1*, *DRB1* full-length, and *DRB2* full-length sequences, were amplified using a pair of primers designed to introduce a 5′ CACC overhanging sequence to allow their directional cloning into pENTR/D-TOPO^®^ (Invitrogen).

The *DRB1* promoter region, containing the 5′UTR of *DRB1* and 538 nt genomic sequence, as previously described (Curtin et al., 2008), was modified to also include the first exon and intron of the *DRB1* gene. The longer *DRB1* promoter region was amplified from genomic DNA using oligos that added 5′ *Sph*I and 3′ *Sal*I overhanging restriction sites. The PCR-amplified sequence was digested and ligated into a modified pEN::L4-2-R1 (also obtained from Plant Systems Biology, VIB). The pEN::L4-2-R1 vector originally encodes the *cauliflower mosaic virus* (*CaMV*) 35S promoter; thus, to remove the 35S promoter, pEN::L4-2-R1 was amplified using oligos specific to its L4 and R1 *att* site pairs oriented to amplify the entire vector except the 35S promoter.

The modified linear vector was digested with *Spe*I (restriction site included via PCR) and re-circularized, resulting in an L4/R1-containing vector that contained a multiple cloning site (MCS), which was also included via PCR. This vector was then digested with *Sph*I and *Sal*I and ligated with the longer *DRB1* promoter sequence, generating the pEN::L4-*DRB1pro*-R1. Finally, gateway cloning was performed using the entry vectors pENTR/D-TOPO containing a chimeric gene, pEN::L4-*DRB1pro*-R1, pEN::R2-GStag-L3, and the modified destination vector, pKCTAP. The resulting expression vector was used to transform *drb1* mutant plants via Agrobacterium-mediated transformation using the floral dip method (Clough and Bent, 1998). Plants were selected for resistance to the herbicide glufosinate. The sequences of oligos used are listed in Supplementary Material.

### RNA Isolation

For all RNA analyses, total RNA was isolated using TRIzol Reagent (Invitrogen) according to the manufacturer’s instructions.

### Real Time RT-PCR

Synthesis of cDNA for real-time reverse-transcription PCR (RT-PCR) was performed using SuperScript^®^ III Reverse Transcriptase (Life Technologies) following the manufacturer’s instructions. RT-PCR was performed using Brilliant III SYBR^®^ MM according to the Agilent Technologies protocol. The sequences of oligos are listed in Supplementary Material.

### miRNA Real Time RT-PCR

RT-PCR for the quantification of miRNA accumulation was performed according to a previous report (Chen et al., 2005). SuperScript^®^ III Reverse Transcriptase (Life Technologies) and Brilliant III SYBR^®^ MM (Agilent Technologies) were used to perform the cDNA synthesis and RT-PCR, respectively, following the manufacturers’ instructions. *Arabidopsis SnoR101* was used to normalize the miRNA accumulation. The sequences of primers are listed in Supplementary Material.

## Results and Discussion

### Expression of Chimeric Gene Series in *drb1* Plants

*Arabidopsis* plants defective in the activity of DRB1—*drb1* knockout mutant plants—exhibit pleiotropic developmental defects characterized by reduced overall size and hyponastic rosette leaves (Lu and Fedoroff, 2000; Wu et al., 2007). To investigate the structural components that direct the functional activity of DRB1, and that differentiate it from DRB2 in the *Arabidopsis* miRNA pathway, we transformed *drb1* plants with a series of chimeric vectors. Each vector harbored coding sequences for DRB1 and DRB2 as full-length, truncations or rearrangements. The *in vivo* expression of these chimeric vectors, termed DRB-C1 to DRB-C9, was driven by the native DRB1 promoter. Homozygous plants were identified in the T2 generation and further selected by gene expression of chimeras relative to endogenous *DRB1* in wild-type *Arabidopsis* (Col-0). The assessment of each chimera’s expression level was performed such that all selected transgenic plants had chimeric genes at levels similar to endogenous DRB1 in wild-type plants. Transgenic plants were then visually assessed for comparison to wild-type and *drb1* plants.

**FIGURE 1:**
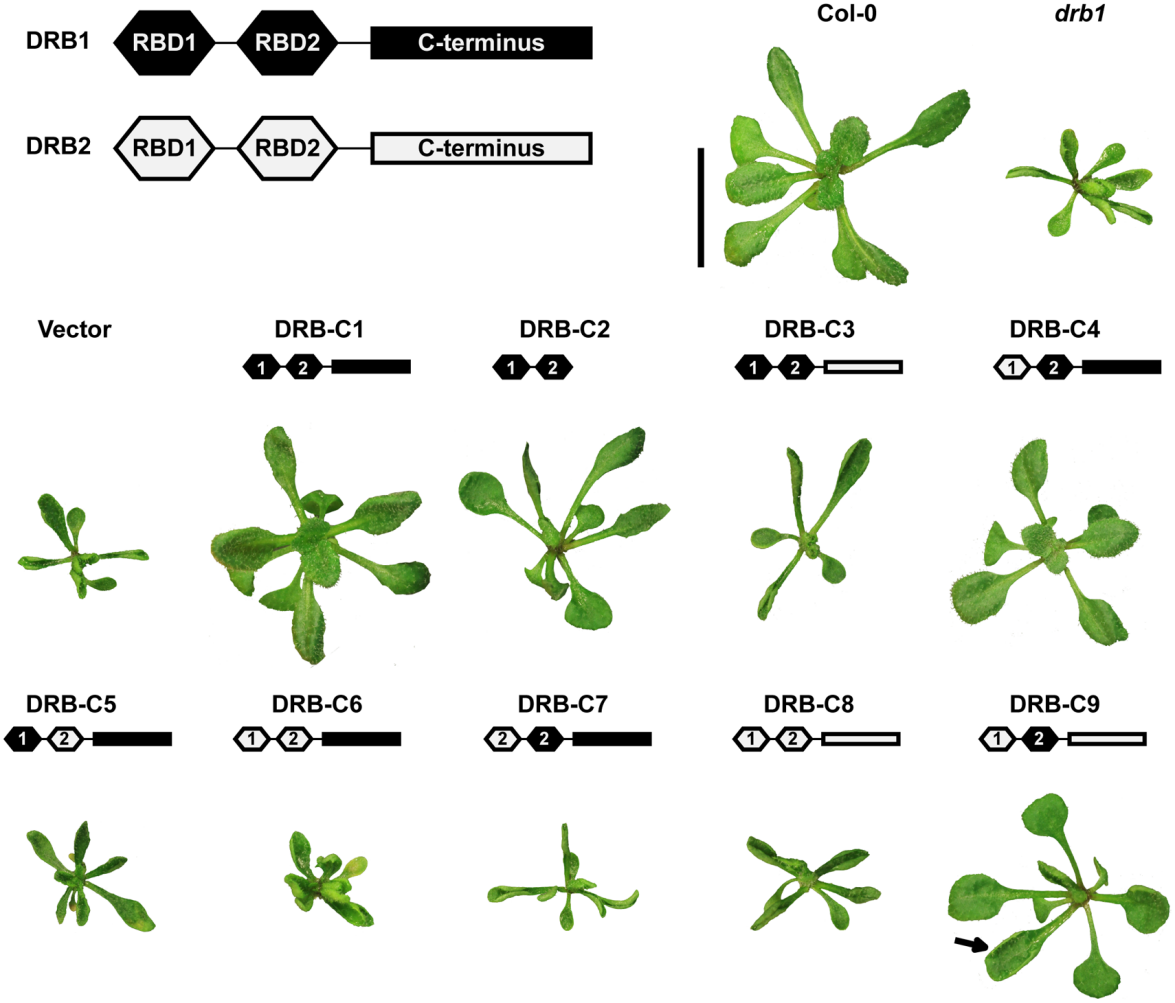
**Phenotype and ABA response of wild-type, *drb1* and transgenic plants.** Col-0 wild-type, *drb1*, and *drb1* transformed with either DRB1 (DRB-C1), dsRNA binding domains of DRB1 (DRB-C2), or a chimeric gene (DRB-C3 to DRB-C9), driven by the endogenous promoter sequence of *Arabidopsis DRB1*. Protein domains of DRB1 and DRB2, used to construct the vectors for transformation, are depicted in the boxed schematic (top), and domain identities of each vector are shown above each transgenic plant. The arrow (DRB-C9) points to a *drb1*-like hyponastic leaf. Transgenic plants were cultivated on MS medium under long-day conditions for 16 days. Scale bar, 50 mm. Approximately 10 homozygous transgenic plants were analyzed for each construct. All transgenic plants expressing the empty vector or DRB-C3, C5-C8 failed to complement the *drb1* phenotype. Transgenic plants expressing DRB-C1, C2, C4, and C9 showed wild-type phenotype in 10 out of 10 plants (10/10), 9/9, 9/10, 9/11, respectively.

Previous work has shown that *in vivo* expression of both N-terminal dsRBDs of DRB1 lacking its C-terminal region, when driven by the constitutive CaMV 35S promoter, was sufficient for complementation of the severe *drb1* developmental phenotype (Wu et al., 2007). Here, we observed that the expression of DRB1 full-length (DRB-C1) or DRB1 N-terminal dsRBDs (DRB-C2), driven by the *DRB1* endogenous promoter in a *drb1* mutant background (*drb1*/DRB-C1 and *drb1*/DRB-C2 plants), also allowed for phenotypic complementation of this mutant (**Figure 1**). This further confirms that the N-terminal dsRBDs fulfill the function of the whole DRB1. However, transformation of *drb1* mutant plants with chimeric genes lacking the non-canonical DRB1 dsRBD2 (DRB-C5, C6 and C8) failed to complement the severe phenotype. Transgenic lines *drb1*/DRB-C5, *drb1*/DRB-C6 and *drb1*/DRB-C8 displayed the *drb1*-like phenotype, showing that the non-canonical domain of DRB1 is essential for its *in vivo* activity. This also revealed that *DRB2* under control of the endogenous promoter of *DRB1* (*drb1*/DRB-C8 plants) is not capable of complementing *drb1* phenotype, whereas under control of the strong and constitutive 35S promoter, *DRB2* can compensate for *DRB1* null mutation (Eamens et al., 2012). This is in accordance with the lower affinity of DRB2 for DCL1 as compared with that of DRB1 protein (Kurihara et al., 2006), and further suggests that DRB2 can only compensate for DRB1 at elevated levels in order to compensate for its lower affinity.

The DRB-C7 chimeric gene also failed to complement the *drb1* phenotype. In the DRB-C7 chimeric gene, the canonical first dsRBD of DRB1 was replaced with DRB2 dsRBD2, which was predicted to fold into a canonical RBD (**Figure 1**). *drb1*/DRB-C7 plants showed a *drb1*-like phenotype, suggesting that (i) DRB2 RBD2 is a non-canonical dsRNA binding domain or (ii) it mediates specific protein–protein interaction(s) different to those of DRB1. DRB1 interaction with its partnering proteins has been shown to require its second dsRNA binding domain (Yang et al., 2010); hence, it is likely that the second dsRNA binding domain of DRB2 plays a similar role in mediating protein-protein interactions.

Although the C-terminal region of DRB1 appears to be dispensable for its function (Wu et al., 2007), the transformation of *drb1* with chimeric genes harboring the DRB2 C-terminal region (DRB-C3 and DRB-C9) resulted in different phenotypes. DRB-C3 has the DRB1 dsRNA binding domains fused to the C-terminus of DRB2, and *drb1*/DRB-C3 plants displayed a *drb1*-like phenotype (**Figure 1**). The DRB-C9 chimeric gene, however, is similar to DRB-C3, with the difference that it has the first dsRBD of DRB1 replaced by DRB2 dsRBD1. Interestingly, the *drb1*/DRB-C9 phenotype was closely related to wild-type, but also had some hyponastic leaves, characteristic of *drb1* mutants. These results show that the DRB2 C-terminus can impair DRB1 function in the absence of DRB2 dsRBD1, suggesting that these domains may interact. In addition, the DRB-C4 chimeric gene has the first RBD of DRB1 replaced by DRB2 RBD1, and *drb1*/DRB-C4 is wild-type in appearance. Although it is possible that certain chimeric combinations may lead to disruption of protein tertiary structure, this is unlikely as several dsRBD chimeric combinations have been previously shown to be functional (Parker et al., 2008; Yang et al., 2010; Lee et al., 2013). However, it is still possible that the chimeric genes that failed to complement the *drb1* phenotype are non-functional because of improper folding, altered posttranscriptional modification (e.g., phosphorylation) or posttranscriptional regulation (e.g., loss of binding site). These are all interesting avenues for further studies, and our results present a comprehensive picture of domain swapping that, due to incompatibility or non-functionality, give combinations impaired in their ability to complement the *drb1* phenotype. These results also suggest that the dsRBD1 of DRB1 and DRB2 are functionally similar in the miRNA pathway.

### Abscisic Acid Treatment of the *drb1* Transgenic Lines

**FIGURE 2:**
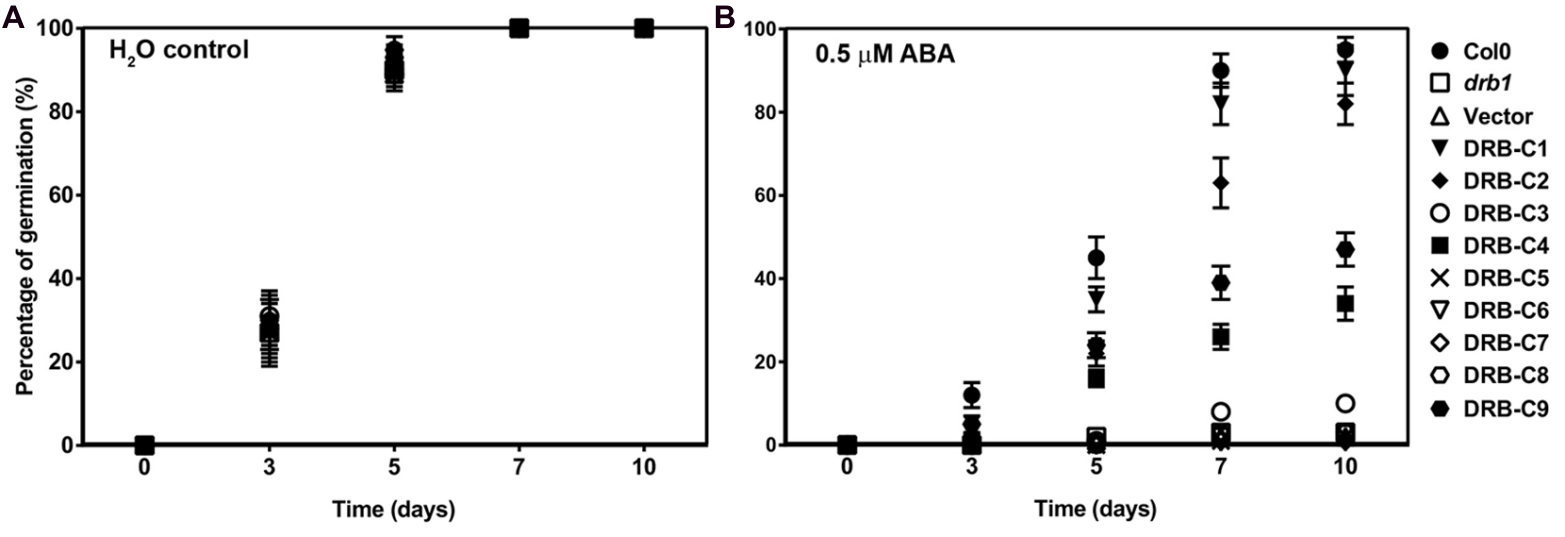
**Effects of exogenous ABA on wild-type, *drb1* and transgenic plants.** Seeds of wild-type, *drb1* mutant and *drb1* transgenic lines were germinated on filter paper saturated with either water **(A)** or 0.5 μM ABA **(B)**. Seeds were incubated at 4°C for 48 h and then transferred to room temperature for assessment of their germination efficiency over a period of 10 days (*n* > 100, ± SD).

It has been previously demonstrated that *drb1* seed germination is inhibited by exogenous ABA application (Lu and Fedoroff, 2000). To further characterize the transgenic populations resulting from the *in vivo* expression of the chimeric vector series, T3 seed was collected from homozygous T2 transgenic lines and the germination efficiency of each seed pool assessed via germination on filter paper soaked with either water or 0.5 μM ABA (**Figure 2**). In contrast to seeds germinated on water-soaked filter paper, on which all assessed *drb1* transgenic lines germinated efficiently, all plant lines showed reduced or completely abolished germination in the presence of ABA (**Figure 2**). In accordance with the phenotypic analysis (**Figure 1**), transgenic lines drb1/DRB-C1, drb1/DRB-C2, drb1/DRB-C4 and drb1/DRB-C9, all of which displayed wild-type-like phenotypes, showed limited sensitivity to ABA, as did Col-0 plants (**Figure 2**). However, seeds collected from transgenic lines that displayed *drb1*-like phenotypes, including *drb1*/DRB-C3, *drb1*/DRB-C5, *drb1*/DRB-C6, *drb1*/DRB-C7, and *drb1*/DRB-C8 plants, were highly sensitive to the ABA treatment (**Figure 2**). Furthermore, the ABA-sensitivity displayed by these transgenic lines was highly similar to the ABA hypersensitivity of non-transgenic *drb1* plants (**Figure 2**). Taken together, these results reveal a clear correlation between complementation of the *drb1* phenotype and ABA sensitivity; i.e., *drb1* transgenic lines that complemented the *drb1* phenotype were not hypersensitive to ABA, whereas the transgenic lines that failed to complement the *drb1* phenotype were hypersensitive.

### miRNA Accumulation and Target Gene Expression in Plants Transformed with a Chimeric Vector Series

**FIGURE 3:**
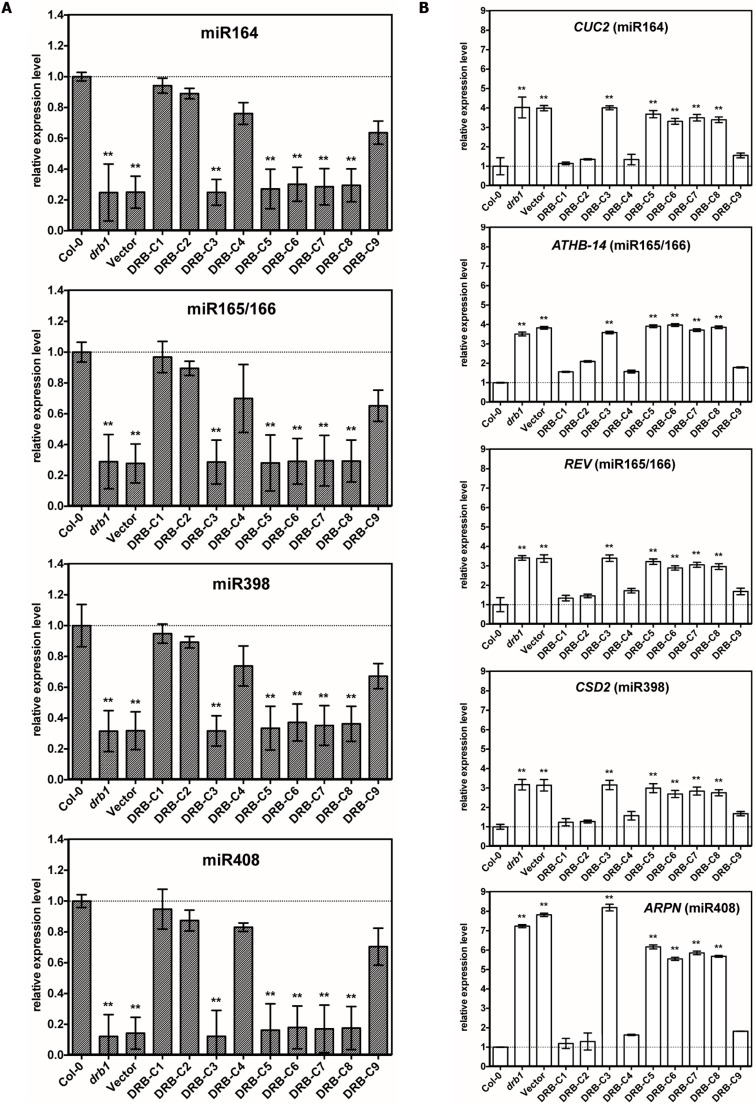
**miRNA accumulation and their target levels in wild-type, *drb1*, and transgenic plants.** **(A)** Stem-loop RT-PCR quantification of miRNA accumulation relative to Col-0 wild-type levels (dashed line) (*n* = 3, ±SD, ^∗∗^*p*-value < 0.01). miRNA levels are normalized to SnoR101 accumulation. **(B)** RT-PCR quantification of miRNA target gene expression relative to Col-0 wild-type levels (dashed line) (*n* = 3, ±SD, ^∗∗^*p*-value < 0.01). Gene expression levels normalized to *ACTIN2* (AT3G18780) expression.

At the molecular level, *drb1* mutants are characterized by reduced miRNA accumulation and de-repression of miRNA target gene expression (Han et al., 2004; Kurihara et al., 2006). The accumulation of four highly conserved and well-characterized plant miRNAs, and the expression of their target genes, was therefore assessed in *drb1* transgenic lines expressing the chimeric vector series. As observed for the ABA sensitivity assay, levels of miRNA accumulation and miRNA target gene expression were strongly correlated with the phenotype displayed by each of the *drb1* transgenic lines (**Figure 3**). In the transgenic lines *drb1*/DRB-C1, *drb1*/DRB-C2, *drb1*/DRB-C4, and *drb1*/DRB-C9, which displayed wild-type-like phenotypes, accumulation of miR164, miR165/166, miR398 and miR408 and target gene expression of *CUP SHAPED COTLEDONS2* (*CUC2*; miR164), *ARABIDOPSIS THALIANA HOMEOBOX PROTEIN14* (*ATHB-14*; miR165/166), *REVOLUTA* (*REV*; miR165/166), *COPPER/ZINC SUPEROXIDE DISMUTASE2* (*CSD2*; miR398), and *PLANTACYANIN* (*ARPN*; miR408) were at approximately wild-type levels (**Figures 3A,B**). Furthermore, the degree of *drb1* phenotype complementation displayed by *drb1*/DRB-C1, *drb1*/DRB-C2, *drb1*/DRB-C4 and *drb1*/DRB-C9 transgenic lines (**Figure 1**) was supported by the molecular analyses presented in **Figures 3A,B**. *drb1*/DRB-C1 transgenic lines that expressed the full-length DRB1 transgene and displayed the highest degree of complementation (**Figure 1**) gave miRNA accumulation and target gene expression levels equivalent to those determined for wild-type plants. *drb1*/DRB-C9 transgenic lines, which expressed the DRB-C9 chimeric vector that housed only the second dsRBD of DRB1, and developed rosette leaves with mild hyponasty, had only slightly reduced miRNA accumulation and a corresponding mild elevation in miRNA target gene expression. Furthermore, *drb1* transgenic lines that expressed chimeric vectors DRB-C3, DRB-5, DRB-C6, DRB-C7 or DRB-C8, and displayed *drb1*-like phenotypes, gave miRNA and target gene expression levels equivalent to those observed in non-transgenic *drb1* mutant plants. Taken together, the phenotypic and molecular analysis (**Figures 1–3**) showed that (i) DRB1 and DRB2 dsRBD1 are functionally similar, (ii) their dsRBD2s appear functionally distinct, and (iii) their C-terminal regions appear to perform different functions.

### Evolutionary Conservation of DRB1 and DRB2

**FIGURE 4:**
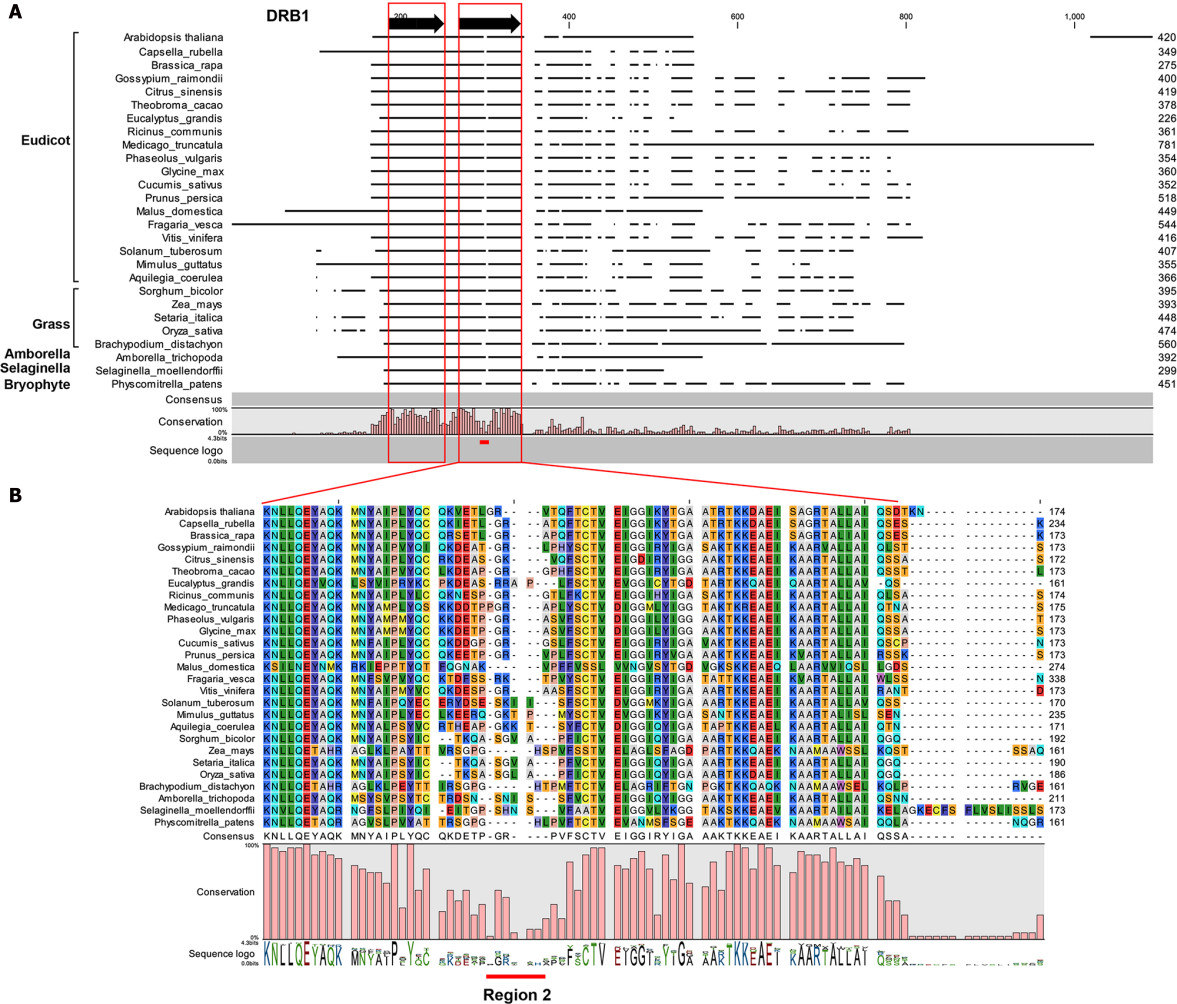
**Evolutionary conservation of DRB1.** **(A)** An overview amino acid sequence alignment of *Arabidopsis* DRB1 (used as the search query) and orthologs identified in other plant species. The regions corresponding to the two dsRBDs of each analyzed DRB1 are indicated by the red boxes. Region 2, which has low sequence conservation in dsRBD1 of DRB1, is indicated by the solid red line. **(B)** Alignment of the amino acid sequence of the dsRBD2 domains of each plant DRB1. The solid red line identifies region 2, the region of low sequence conservation in dsRBD2 of DRB1.

**FIGURE 5:**
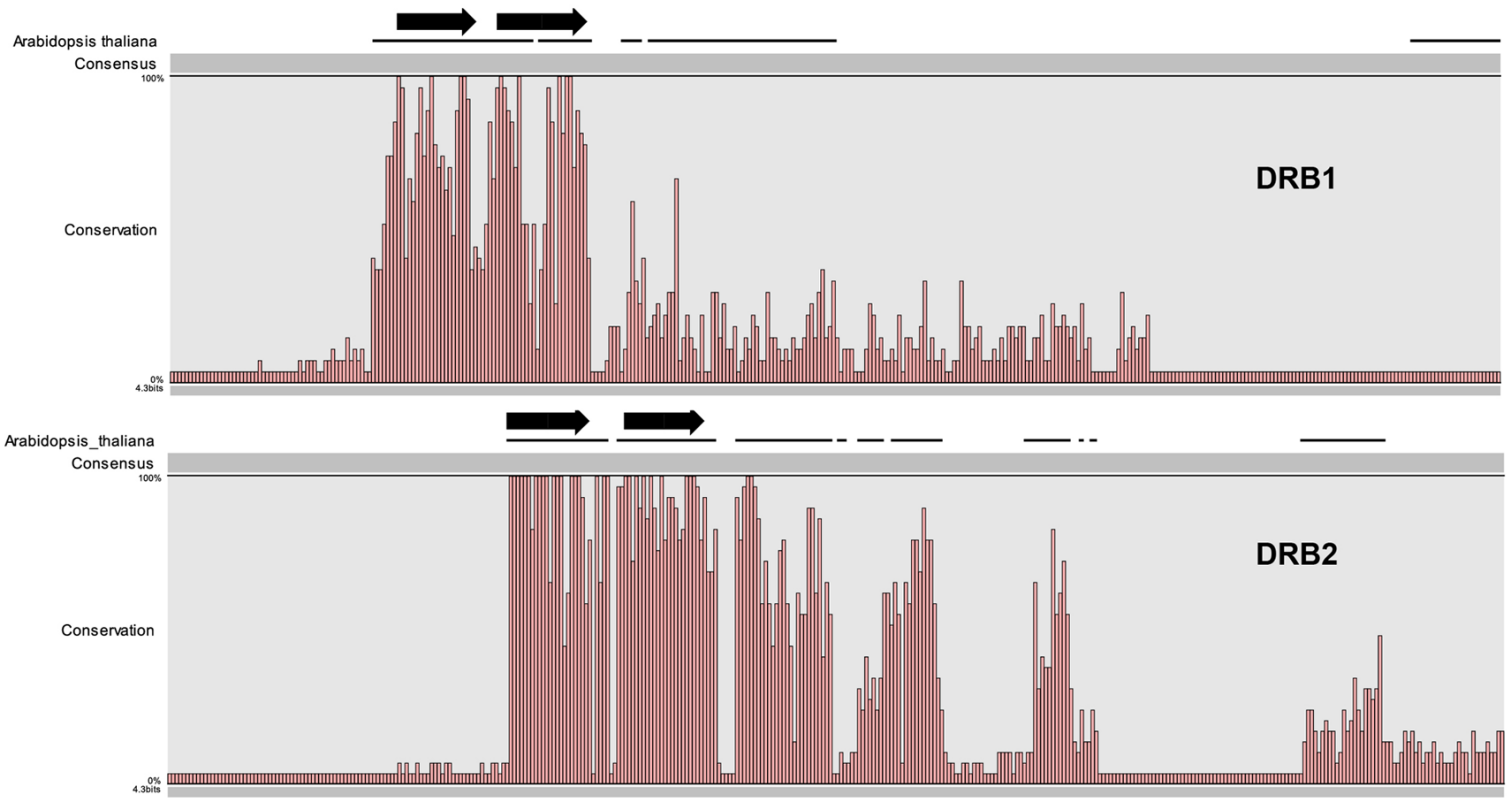
**Comparison of the conservation between identified DRB1 and DRB2 orthologs.** The full-length sequences of DRB1 and DRB2 orthologs were scaled to the same size for direct comparison and the level of amino acid sequence conservation across these two DRB proteins schematically shown.

DOUBLE-STRANDED RNA BINDING1 and DRB2 play a major role in determining the silencing fate of a miRNA (Reis et al., 2015). Here we identified the structural components of DRB1 and DRB2 required for the miRNA-guided transcript cleavage pathway. To further characterize these proteins, we studied their evolutionary conservation among land plants. The *Arabidopsis* DRB1 sequence was aligned to those of orthologs identified in public databases. The DRB1 orthologs were from a wide range of evolutionarily diverse plant species, including eudicots, monocots, and a moss. The alignment clearly showed a high degree of conservation in the N-terminal region relative to the whole sequence (**Figure 4**). Previously it has been shown that the severe developmental phenotype of *Arabidopsis drb1* plants can be complemented via the transgene-based expression of a truncated version of *Arabidopsis* DRB1 (419 aa) lacking the 249 amino acid residues of the C-terminal domain (Wu et al., 2007; Yang et al., 2010). This is in accordance with our amino acid alignment because of the lack of C-terminal conservation, suggesting that the DRB1 C-terminus may not be required in all plant species. A lack of sequence conservation is also evident in region 2 of the second dsRBD of DRB1, dsRBD2. In canonical dsRNA binding domains, region 2 is characterized by a GPxH motif (where x is any residue), which is crucial for recognition of, and binding to, molecules of dsRNA (Gleghorn and Maquat, 2014). The dsRBD2 of *Arabidopsis* DRB1 has been shown to be a weak, non-canonically structured binding domain that primarily mediates protein–protein interaction, and not dsRNA recognition or binding (Yang et al., 2010, 2014). The lack of sequence conservation within region 2 may therefore account for the function of dsRBD2 in DRB1 in the miRNA pathway among land plants. The sequence conservation of DRB2 orthologs in land plants revealed two surprising findings: i) both DRB2 dsRBDs have very high sequence conservation, and ii) compared to the C-terminal region of plant DRB1 orthologs, the C-terminal region is markedly conserved (**Figure 5**). Taken together, these results suggest that DRB1 dsRBD2 has a conserved low affinity for dsRNA and that DRB2 has been more conserved in evolution than DRB1.

### *Arabidopsis* DRB dsRNA Binding Domains

**FIGURE 6:**
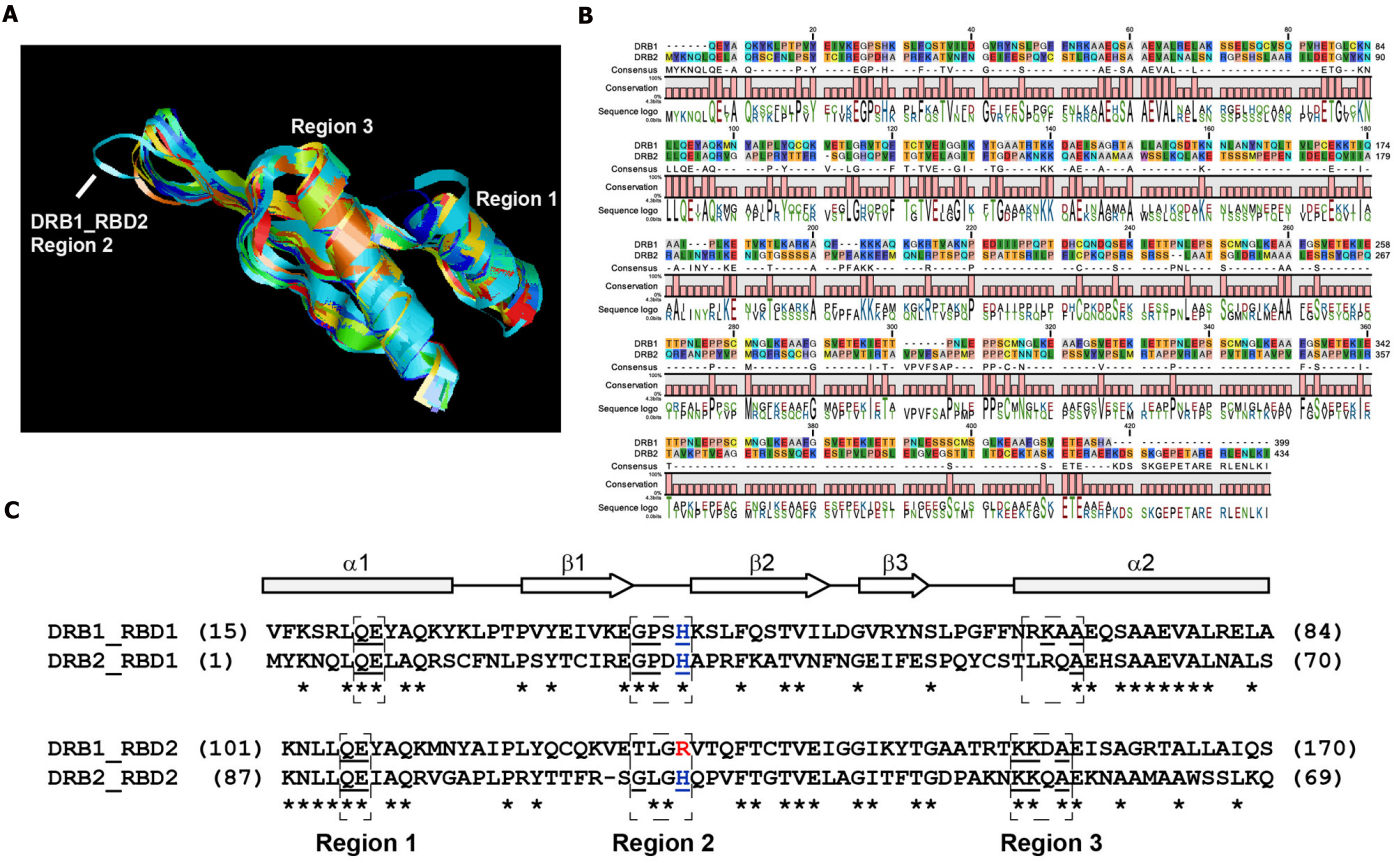
**dsRNA binding domains of *Arabidopsis* DRB proteins.** **(A)** Alignment of dsRBD structures of DRB1 (3ADG and 3ADJ) and predicted structure of DRB2, DRB3, DRB4, and DRB5 RBDs. Region 2 of non-canonical DRB1 dsRBD2 is shown (blue ribbon). **(B)** Amino acid sequence alignment of *Arabidopsis* DRB1 and DRB2. **(C)** Alignment of dsRNA binding domains of DRB1 and DRB2. Secondary folding structure is shown (top). dsRBD regions 1, 2, and 3 are boxed, and their conserved amino acids are underlined. The conserved histidine (H) of region 2 is shown in blue, and arginine (R) in red.

To study whether the lack of canonical structure of DRB1 dsRBD1 is unique to DRB1, the secondary structures for both dsRBDs encoded by *Arabidopsis* DRB2, DRB3, DRB4, and DRB5 were predicted and superimposed onto the crystal structures of these domains of DRB1. This *in silico* analysis showed that dsRBD1 and dsRBD2 of DRB2, DRB3, DRB4, and DRB5 appear to adopt the canonical structure of DRB1 dsRBD1 (**Figure 6A**). The domain superimposition also revealed that region 2, the loop between β1 and β2, was only re-oriented in DRB1 dsRBD2. This structural alteration to dsRBD2 has been previously suggested to reduce the dsRNA binding affinity of DRB1 (Yang et al., 2010). We then analyzed the amino acid sequence of DRB1 and DRB2 in more detail to gain insights into the secondary structures of these proteins. Although both DRB proteins are required in the miRNA pathway, their amino acid sequences are strikingly different, suggesting that they display different surfaces for protein–RNA and protein–protein interactions (**Figure 6B**).

In a canonical dsRNA binding domain, regions 1 and 2 bind dsRNA minor grooves while region 3 binds the dsRNA major groove (Gleghorn and Maquat, 2014). Region 1 is conserved in both dsRBDs of DRB1 and DRB2 (**Figure 6C**). Region 3 of DRB1 dsRBD2 and DRB2 dsRBD2 is also tightly conserved, but is dissimilar in their first dsRNA binding domain. Region 2, in contrast, has the conserved GPxH motif in the dsRBD1 domains of both DRB proteins, but was only present in the dsRBD2 of DRB2. This region aids dsRNA binding to the major groove via the histidine (H) residue of the GPxH motif, which is absent only in DRB1 dsRBD2 (**Figure 6C**). Nevertheless, when this residue was mutated in DRB1 dsRBD1, a dsRBD with high affinity to its dsRNA substrate, only a slight decrease in dsRNA binding affinity was observed (Yang et al., 2010). Taken together, these analyses provide evidence that DRB1 and DRB2 differ substantially at the amino acid and secondary structure levels.

## Conclusion

**FIGURE 7:**
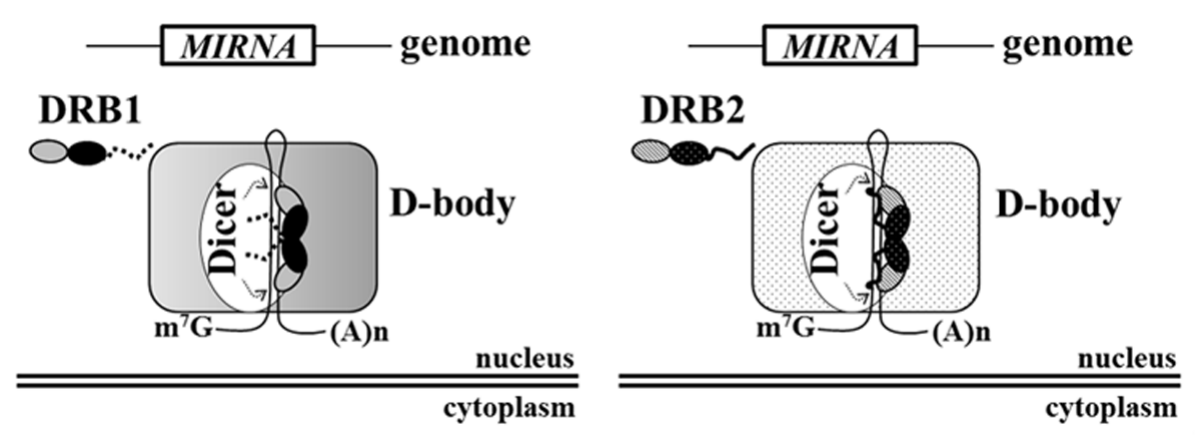
**Hypothetical model for the role of DRB1 and DRB2 domains.** miRNAs are encoded as gene-like structures and are recognized by a dicer (DCL1) and a DRB protein. DRB1 dsRBD1 recognizes dsRNAs and its dsRBD2 promotes dimerization and interaction with other proteins, without the aid of its C-terminus (shown as a dotted line). DRB2 dsRBD1 also recognizes dsRNAs and its dsRBD2 appears to mediate different protein–protein interactions to those of DRB1, possibly assembling dicing bodies (D-bodies) different to those containing DRB1. This may also require the presence of the DRB2 C-terminus (shown as a continuous line).

Although our knowledge of the biogenesis of plant miRNAs has improved dramatically in recent years, several of the latest findings indicate that some important mechanisms remain poorly understood. The biogenesis of miRNA/miRNA^∗^ from miRNA-containing intermediates occurs in dicing bodies (D-bodies), and a growing number of genes, in addition to well characterized core components (e.g., DCL1, SE, and DRB1), have been shown to be required in this process (reviewed by Rogers and Chen, 2013). Thus, it is likely that the D-bodies are dynamic and may vary in protein composition according to developmental stage, environmental conditions and even precursor transcript structure. Although DRB1 is a well-characterized DCL1 partnering protein, our results reveal that DRB2 has been much more conserved during plant evolution. In addition, DRB1 and DRB2 have similar but functionally different domains, such as their dsRBD2 and C-terminus. The results presented here, together with our previous report (Reis et al., 2015), suggest that DRB1 and DRB2 act as bridging proteins in the assembly of different component proteins, and even different RNAs, into the core of the D-bodies, thus altering the properties of the D-bodies and the functionality of the miRNA pathway as a whole (**Figure 7**).

## Conflict of Interest Statement

The authors declare that the research was conducted in the absence of any commercial or financial relationships that could be construed as a potential conflict of interest.
