# Supplementary material for: Chimeric DCL1-Partnering Proteins Provide Insights into the MicroRNA Pathway
Source: Front Plant Sci. 2016 Jan 6;6:1201. doi: 10.3389/fpls.2015.01201 (PMC4701949; doi:10.3389/fpls.2015.01201)
Supplement: Supplementary file 1 [file Table_1.PDF]

SUPPLEMENTARY INFORMATION

Chimeric DCL1-partnering proteins provide insights into the microRNA pathway

Rodrigo S. Reis[1,2,3,*], Andrew L. Eamens[4], Thomas H. Roberts[3] and Peter M. Waterhouse[2,5,*]

[1] Department of Plant Molecular Biology, University of Lausanne, Lausanne, Switzerland.

[2] School of Biological Sciences, University of Sydney, Macleay Building A12, Sydney, NSW 2006, Australia.

[3] Department of Plant and Food Sciences, Faculty of Agriculture and Environment, University of Sydney, NSW 2006, Australia.

[4] School of Environmental and Life Sciences, University of Newcastle, Callaghan, NSW 2308, Australia.

[5] Centre for Tropical Crops and Biocommodities, Queensland University of Technology, Brisbane, QLD 4001, Australia.

* Corresponding authors:

Rodrigo S. Reis (E: rodrigo.siqueirareis@unil.ch)

Peter M. Waterhouse (E: peter.waterhouse@qut.edu.au)

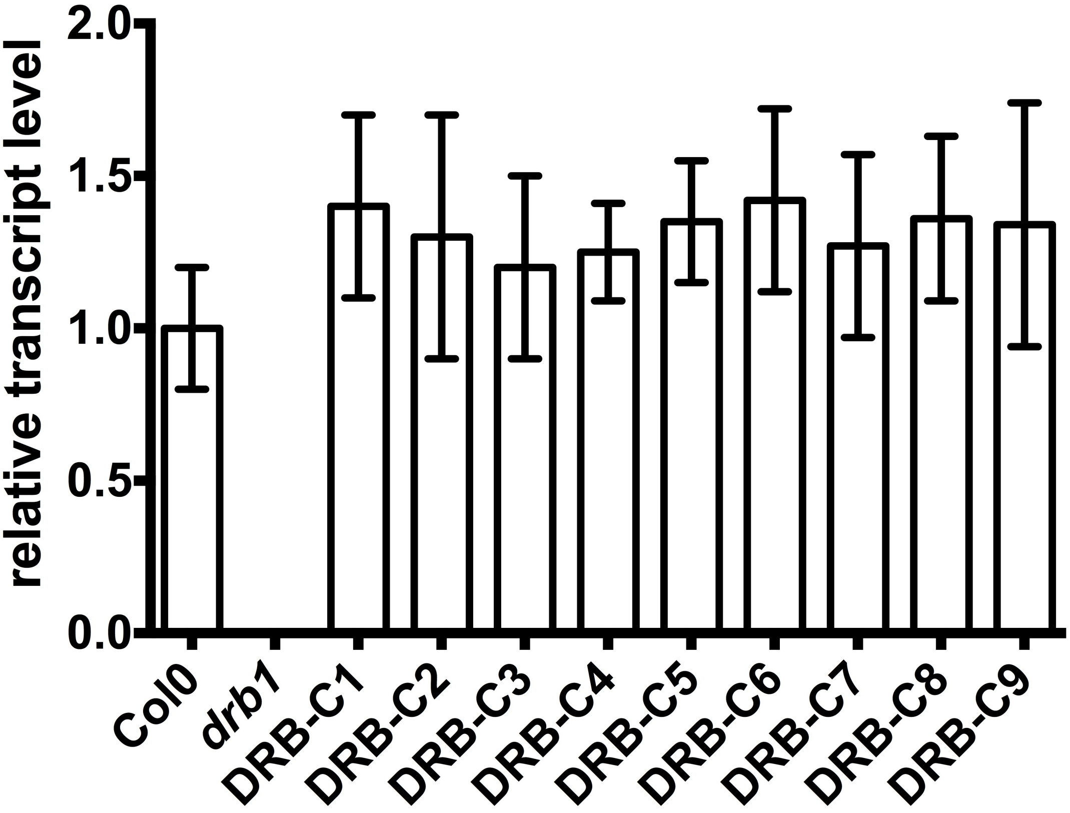

Supplementary Information 1 Expression levels of chimeric genes relative to *DRB1* expression in Col0 wild-type plants (n = 3, ±SD). Gene expression levels normalized to *ACTIN2* (AT3G18780) levels.

Supplementary Information 2 List of *Arabidopsis* DRB1 and DRB2 orthologs.

Plant species [locus identifier].

>DRB1_Arabidopsis_thaliana[AT1G09700]

MTSTDVSSGVSNCYVFKSRLQEYAQKYKLPTPVYEIVKEGPSHKSLFQ STVILDGVRYNSLPGFFNRKAAEQSAAEVALRELAKSSELSQCVSQPVHETGL CKNLLQEYAQKMNYAIPLYQCQKVETLGRVTQFTCTVEIGGIKYTGAATRTK KDAEISAGRTALLAIQSDTKNNLANYNTQLTVLPCEKKTIQAAIPLKETVKTL KARKAQFKKKAQKGKRTVAKNPEDIIIPPQPTDHCQNDQSEKIETTPNLEPSSC MNGLKEAAFGSVETEKIETTPNLEPPSCMNGLKEAAFGSVETEKIETTPNLEPP SCMNGLKEAAFGSVETEKIETTPNLEPSSCMNGLKEAAFGSVETEKIETTPNLE PPSCMNGLKEAAFGSVETEKIETTPNLESSSCMSGLKEAAFGSVETEASHA

>DRB1_Aquilegia_coerulea[Aquca_007_00512]

MSITTDFPSMSNCFVFKSRLQEYAQKAGFVTPVYETVKEGPSHEPCFSS SVIVDNVKYNSLPGFFNRKAAEQSAAEVALLELAKSGKLNESISCPVHETGLC KNLLQEYAQKMNYALPSYVCRTHEAPGKKTSYFCTVDIGGIQYIGATAPTKK EAELKAARTALLAIQTNTSRAESNPNGSHELTVLPCKRKGTEPDAEKETPQPM KPKKTKFKKSKKQRSLQNRIDLATGNMESSLVLQGEDTRMLEAIKDEHGGEH LRSQVNEYEQVPITDPNNNSQNEQTFPLNSNPSDQGYLNMHLDSGVQVKLEN LVNVVGAEQVATFGAAEIPVVNGEKFDSDNSLVGLNQTEDVRSEVKVEHGV GGC*

>DRB1_Amborella trichopoda[AMTR_s00033p00230080]

MLCIKGWYFNAHKVGFDNLADLKTLSVHHLFLAEKLSLMSIRRGCML EGGVSNCYVFKSRLQEYAQKAGIPTPVYETVKEGPSHEPIFRSSVTINDVKYD SLPGFANRKAAEQSAAEVALMELLKSGTMDCIPHPVHETGLCKNLLQEYAQ KMSYSVPSYTCTRDSNSNISSFVCTVEIGGIQYIGGAAKTKKEAEIKAARTALL

AIQSNNSETSNSGSVYTVLPGNKKEKVSDGRSETPAPLKTKKKKSKFKKGSQ

RRHPRFKGGLKVSENEQEKLGPTDGSIVKTEGFGDVVGAEARANDREHLKPD

EAVTRENGERCHQLEHETEKASTDRDIPNGLKGFDHTPISVEPLGAQTSSVNG

DLGERVEINSKGQGAAMEGLPGVTPNLSED

>DRB1_Brassica_rapa[Bra019999]

MTANEVSSGVSNCYVFKSRLQEYAQRYKLPTPLYETIKEGPSHKPLFQ

STVIVNDVRYDSLPGFFNRKAAEQSAAEVALQELAKSSDLTQSVSLPVHEMG

LCKNLLQEYAQKMNYAIPLYQCQRSETLGRAPQFTCTVEIGGIKYTGAATKT

KKEAEISAGRTALIAIQSESKMDLANNYSTQLTVIPCEKKTVEVASPVKETIIK

TPKARRAQFKKKARKGKLKVAKDLEDSTIPPQPTEHCQNQQLNLEPSSCVNG

FKEAAFASVETEASQA*

>DRB1_Brachypodium_distachyon[Bradi2g35730]

MYKNQLQELAQRSCFNLPSYACIREGPDHAPRFKATVNFNGESFESPT

FCSTLRQAEHAAAEVALNELSKRGPSSSLAAKVLDETGIYKNLLQETAHRAG

LKLPEYTTIRSGPGHTPMFTCTVELAGRIFTGNPGKTKKQAQKNAAMAAWSE

LKQLPRVGEAASSSPLDHDDEEQEQVIVARTLESLNQTNEGKSAHQKEKQQS

NNHPPPRRSHPKPYVSFQRSRLQNQTYSNVSPEQAMYHWRQVQPTQQKPHFP

MVPVMGNTRFPSPPTMLSMYPPPPQGQFPVPASQDNLALLPCFREAASVLPQ

YFSPYPASYVSRSPLPVTVHAMHGERQGCTQMVELPDAVVFSRYAASASSST

SEKGAPSKVQELPENGKDAYTESSAAPEEENRAPQTSSSSTSRSVSQKLEPNK

SKESNKPTEQPPKSSSTRVSSSVVQRPIQRECYPSSIQHVEPIHRSNLPFSMATS

PELWSPHMQAPATPMNTSGSFYEQRPHWYAAPVTVRTAIPVCSARPNAGSSS

AGAQPAVRILSRNEPEAHRNTRDTSKAATASLELNKLHI*

>DRB1_Capsella_rubella[Carubv10009604m.g]

GRQDTEERERSRGGRVFGEGLKSCLSLGASSQHHTHAVPRNKFLFLAL

PILLLSYILLVKMTTANDLSTGVSNCYVFKSRLQEYAQKYKLPTPIYETVKEG
PSHKAFFHSTVILDGVRYNSLPWFLNRKAAEQSAAEVALQELAKSSDPSQCV
SQPVHETGLCKNLLQEYAQKMNYAIPLYQCQKIETLGRATQFTCTVEIGGIKY
TGAATRTKKDAEISAGRTALLAIQSESKKLANYNTELTLLNFNTQLTVLPFDR
KSFEAVIPVKETIKTPKSRKAHFKRKARKGKRKVAKDGEDMLIPPQPTEHCQ
NDQSEKIETTLNLEPSLCMNGGKEAAFGSVDTEASQA*

>DRB1_Carica_papaya[evm.TU.contig_34457.2]

HETGLCKNLLQEYAQKMNFAIPLYQCQKEEAPDRMPCFSCTVEIGGIR
YIGAVAKTKKEAEIKAARTALLALQSSRDKSGSSQLTVLPCKKRAGEPVNSTE
EVTNVTKAKKARVKKLSKKRFRGKKVEQDQVNNTGSSDASVVQVTDSGAL
ASKQATSNCEQDLDLSEDKVASPEGVCPPFSTHKSQNENSRKENSGIGNHKTP
DVGTSAISFADTTAIDEEVNGVTVVTEVASVVSNSPLGRIEA*

>DRB1_Citrus_sinensis[orange1.1g014809m.g]

MPTNEGFSGVSNCYVFKSRLQEYAQKAGIPTPVYETIKEGPSHEPWFR
STVIVDDVRYDSLPGFFNRKAAEQSAAEVALVELAKLGKVNECISQPIHETGL
CKNLLQEYAQKMNYAIPLYQCRKDEASGKVQFSCTVEIGDIRYIGGAAKTKK
EAEIKAARTALLAIQSSASELSGNSAGNTQLTVLPSRKRGPEVANNPEETVNV
PKAKKGRFKKKILKMKRPGGRMDRTQLQNTGNMENINASQEGSKVSQANTS
GIQGVSTEVLAVEGTMDCQEGRSEIEPIEREMPAVNDALPHHIGGDSETIHSA
ATHCSDRSSNEASEMGTSSLAKEVNEVTPGVGTSSVSCHTTALAKEVNEGTP
NEGTSTALTEEVNEPSGIMEAASMTNNSTLGQIESSCVIPGINQPEERIQAGTSQ
A*

>DRB1_Cucumis_sativus[Cucsa.160550]

MPAKDNFQGVSNCYVFKSRLQEYAQKVGLPTPVYETIKEGPSHEPTFH
STVIVNDVRYDSLPGFSNRKAAEQSAAEVALMELSKSSDLNPHVSQPVHETG

LCKNLLQEYAQKMNFAIPLYQCQKDDGPGRGSLFSCTVEIGGIRYIGAVAKT

KKEAEIKAARTALLAIQSCPNSLSEKSVNQVQLTVIPSCKRKEAADCSVKPKS

TASPRAKKGRFKRFKRGVLRNRDFINGLVNLDFDNTDRSTLEPFTTGVVQLP

GYVGPVDLAKDTLLNSECRTTDLSSNNNDVLVSNVQSDMPLLLNGNSGNGC

SGTFNSNQVNCATSNVMSSPLTDVQPTCSEVANVAAGVTGELQ*

>DRB1_Eucalyptus_grandis[Eucgr.B01706]

VSNCNLFKSRLQDYAHKTGLPTPLYETIKEGALHESQFRSTVTVNGVK

YNSLPGFCNRKAAEQSAAEVALLELAKSGEISEGISQPVHETGLCKNLIQEYV

QKLSYVIPRYKCPKDEASRRAPLFSCTVEVGGICYTGDTARTKKQAEIQAART

ALLAVQSSTPTDSQLTVPSKKRATETSPFNVRQSSPENSTMNQNGEDDAAQE

QLSLRRSALIRARQNPQCF*

>DRB1_Fragaria_vesca[gene12149-v1.0-hybrid]

MVLSIEVSSIFLSSARLRLQRLPPSPAPAPLLPLPENEMSGLLSQPEMVP

KDKASFWLLELGKGTDEKKEPRQSTWHPTLLRSFSLLRFLSFYLSTSTSSARPS

SPLLLLLRLRPPIKDALHLESPIAACLRRLNRIGVYSSNTGFPERQTIWVSFCQF

GGHFKVMAKSEEFQGVSKCYVFKSRLQEYAQKMGIKIPVYETTKEGPSHEPS

FRSTVVVNDVRYDSLVGFTHRKAAEQSAAEVALRELSKSGDAAKCISQPVQE

IGLCKNLLQEYAQKMNFSVPVYQCKTDFSSRKTPVYSCTVEIGGIRYIGATAT

TKKEAEIKVARTALLAIWLSSNESSMGSFETSELTVLPSKKRRLESEEASNKPK

RKKVPFSRKRMLKRKPNEENAVPGLESNEAMPQDSVSEDIPHANNVYAEHGP

STASDLTNGSNGNGFVGCGDLTTLAKEVNMVSAVKPMTAEAMPQDSVFEHI

PHANNVNAEHGLSTTSDFNATNGSCAVGDSISPSSENTTTLAKEGNMVSAGP

YLEASSVMPGPESLDQSQ*

>DRB1_Glycine_max[Glyma04g10230]

MPTNEDFQSVSNCYVFKSRLQEYAQKAGLPTPVYETIKEGPSHEPSFRS

TVIVNDVRYDSLPGFFNRKAAEQSAAEVALVELVKSNAVNQSITQPVHETGL
CKNLLQEYAQKMNYAMPMYQCKKDETPGRASVFSCTVDIGGILYIGGAAKT
KKEAEIKAARTALLAIQSSASHASENQVGHPQLTVLPCRKRATSSVAIADENS
NPPKPKKARFKRKSSKRKPSRDKMGQIHTENLGIGEIINHEVETHASVNGESG
LLEKKTETFTSEATNNFENGVSVNYQEKETLAGESSFAVNSQEGIFENGKSTE
LYSKENNLGTVVTELSSASNGGIPEPSVEVNKQQCNGEMVSDHCVVGD*

>DRB1_Gossypium_raimondii[Gorai.010G034400]

MPTNENFSGVSNCYVFKSQLQEYAQKVGLPTPVYETFKEGPSHKPSFR
STVTVNNVKYDSIPGFFNRKAAEQSAAEVALLELFKAGEANESISQPVHETGL
CKNLLQEYAQKMNYAIPVYQIQKDEATGRLPHYSCTVEIGGIRYIGASAKTK
KEAEIKAARVALLAIQLSTSQLTDRALGNSQLTVIPCRKRAIETASNPDETTVK
APKPKKSRFKKKMLKTKFLGKKADRSQDNPTSNSAIGSDDSHKPESIQTDSFT
ALGSETLGTEATNNLKDAKITSDSSEREMPSADVALAPEVADNSKNEQLMAA
NPLHSNHEVPDVENSSMVCDDQTDSVKLTNGDDVASKITDPTSSQMEASKIM
PGLNQVVEKVHANAGQAQVLEKIDIGSVPGIQFRN*

>DRB1_Linum_usitatissimum[Lus10008862.g]

MSNGLHAGRLPLHASSSSSQQQPTVGKRPRTDNSIASCYLFKSQLQE
YAQKAGVPTPVYETTKEGRSHEPFFRSTVIVNDVRYDSLPGFMNRKAAEQSA
AEVALIELAKSSDANASISQPAHGTGLCKNLLQEYAQKMNFAVPLYQCQQYE
TQHRGRRGHSFQCVVDIGGTRYIGAMANTKKEAEIKAARTAWFAIKEINMDI
SRGPTGGNSSLTVIPNRNRVLEPPSVPKETAAPPTVKKGRRKKNMMKRKRKR
KPQNALGEHSSNGNPAGADLKGLELQHTNMSETEVPKSALNEHINNTSETAD
DQQGSECKRIRVSEPEVEGMKVVTGESVVEGVIGAIGESKVEGMSGAIGDIIV
GGGNEQMDGAIGESKVEGMSGAIGREIVVGGGNERMEGAIGESEVEGVDKQ
MEGAIGESKVEGVDEGMSDAMGEIVLEGGNELMESVIGESNIEGLDEGMSDA

IGEIVLEGRNVLMEGVTGESEVEVDKRTEGAIGESKVEGVDEGMRGAIREIIV
EGGNDRKEGAERESEVGVDKGTEGAIGGSEVEGVDKRKEGSIGESKVEGVDE
VMEVALGGSEVEGVQSGSVVEIVKGALSESIVEGVETALGASNDSVKDSNPT
PENHPEPVLQTCGPLPVEGEGGIQSAVEISAPDSNPTLGNRPEPVLQTGGALPV
EGKGGILSVMEISASTSNEDTATVIGSQAGHAIKSADVGVSGGSDGQSSGCSD
*

>DRB1_Malus_domestica[MDP0000219451]

MEASDSNLSQLQLQLSNQPPPQNPTPVHHQFLNPAPVQDQVPAQHPAS
SASPSASSASASASSSKNEAKTILSMPLHCLGCAGKVFWHYTKHELLTVKSGC
KVAQELERYGLPDHLTHKNRLQEYAQGAGLPLPVYQTVNEGNRHAPSTFRSS
VLVDGVXYASSNTFASRKAAEQDAARLAMISITEKIKDEVPTIPPVHEDLVFC
KSILNEYNMKRKIEPPTYQTFQGNAKVPFFVSSLVVNGVSYTGDVGKSKKEA
EQLAARVVIQSLLGDSESAPIMSEILRSKYKLAHKSKEPDSSHAXLATSAVNT
GQGYVISTDTPVVVAPEASLGMHPPRHEFIKRKQEEPASQAPPQLPIAFVPPAC
KQPLVVVSTSLATTPTSLAPASTSLAPASASLAPASASLAPASTSLXPAPTSLGP
APTFWAVLPASKKRRKNKKSETNLQNDNQ*

>DRB1_Medicago_truncatula[Medtr3g100120]

MSTTNEDFQGVSNCYVFKSQLQEYAQKAGLGTPVYETTKEGPSHEPS
FRSTVIMNDVRYDSLAGFFNRKAAEQSAAEVALMELAKTGEVNQSITQPVHE
TGLCKNLLQEYAQKMNYAMPLYQSKKDDTPPGRAPLYSCTVDIGGMLYIGG
TAKTKREAEIKAARTALLAIQTNASQASENQFGHLTVIPSRKRATESIADEASK
APKSKKSRFKGKYSKKKPHRNKKRRINADNAGDEAKIDNGAESLASANDES
GLQEIKSEAAFPSEVMKNSENGVSSNHHEKETLAGLQEIKSEAAFPSEAMQYY
DNGVSTNHHEKETLAGLQEVKSEAAFPSEAMKNSENGASTNYHEKETSAGL
QEVKSEAAFPPDEAMQNSENGVPTNHHEKETLGAIKEVKSEAAFPSDEAMQN

SENGASTNHHEKETLAAIQEVKSEAAFPSDEAMQNSENGVSTNHHEKETLATI
QEIKSEAAFPSDETMMNSENGVSTNHHEKETLAAIQEIKSEAAFPSDEATMNS
ENGVSTNHHEKETLAAIQEIKSEAAFLSEAMTNSENRVSTNHHEEEKLAGVQ
EIKSEAAFLSEVMKNSGSDISTNLHEKEMYQSFVLNNQENFESGKLTELQSKEI
NIGNVVTEVSFAPEGYILAMSVEMNKQNCNGDMVSGSWWCDSQDSHPHHP
CQFFHRAAPEVFGGYCIAAAITMLLVLFRSFVLVWFLFTTVVSVGSFLGGSKV
AADGFVLIYGRRCRRRPKQCSFCSLRLSQTLSDRLHQHRSVQLRFCVSDPTSY
H*

>DRB1_Mimulus_guttatus[mgv1a009077m.g]

MAESESIFQMEAGEVEIGGVYDQIIGTYYEPATPLGQEEHFGTPRYEPI
VESSAKKRKVNPSNQDSQVKDGPSVSNCFLFKSRLQEFSQKAGFTSPVYETIK
EGRSHEPSFKSTVIVNNVRYDSLPGFFNRKTAEQSAAEVALMKLSNSADMEF
GISQPVHETGLCKNLLQEYAQKMNYAIPLYECLKEERQGKTPMYSCTVEVGG
IKYIGASANTKKEAEIKAARTALISLSENQTSSNSVYTVVPKKKKVSDHGIAA
QEVPTPAVKKPKKRSLKKKMQQQQWKKKRRALKRGTSGEEGTTRLAVNKA
DSVSLTGVASGDVGAVTPHHHDDMSPQVYDRWRSFWCCGGSDPPS*

>DRB1_Oryza_sativa[LOC_Os11g01869]

MDMPPTPLPPETANTSPAPNGATAGIRVENCYVFKSRLQEYAQKAGL
QTPEYHTFKEGPSHEPVFKSTVVINNTSYDSLPGFFNRKAAEQSAAEVALMEI
VKSIPANANIPAVQETGLCKNLLQEYAQKMNYAIPSYICTKSASGLAPFICTVE
IGGIQYIGAAARTKKDAEIKAARTALLAIQGQSEGSANGATKYIVVPGKRVGK
EVEKMPIETPKPLKIKKGGFKKKWNKRKFMKKDGQAVVEKDEARVAGDAH
DSDVLMQPTVITQEASCGTLFLQPCEEAKRVEAEPPRDIEMVQPDKENQHSD
AALVQPDDEARVEQEPSRDISVVPPNEEAISVKQEPSIDAAILQPKEEASSVKQ
EPFIDTAMLQACKEAGSVELGPARDTVISQLNEQDRGVKQEPAGDTAVPQPD

VDARVVKEESPRTEPNGEATNMKETPKNSAVCNSPETKEFGDITAMGSDPPA

TNMSEE*

>DRB1_Phaseolus_vulgaris[Phvul.009G036100]

MPTNDDFQGVSNCYVFKSRLQEYAQKAGLPTPVYETVKEGPSHEPSF RSTVIVNDVRYDSLPGFSNRKAAEQSAAEVALVELAKSNVVNQSITQPVHET GLCKNLLQEYAQKMNYAMPMYQCKKDETPGRASVFSCTVDIGGILYIGGAA KTKKEAEIKAARTALLAIQSSATHASQNQVGHPKLTVLPCRKRVAESVSIADE NSNTPKPKKARFKRKSSKRKNPRDKIGPIHTENVVGIGTNINHEVETQASVND ESGVHEMKSEKFTSEVMKNLENGISFNYPEKEVFAVESSFVFENGKSAELYSK ENNLGSVVAELSSVSNGDILETSVEMNKQQYNGEMVSDHCVVGG*

>DRB1_Physcomitrella_patens[Pp1s344_1V6.1]

MPVPLSQRASEKIDFAMYKSQLQEYAQKQGLMSPSYEYVKEGASHEP RFKSTVWVNGRGYESAPGYPTLRSAEHAAAKAALDFLQKTQFKVVPVHESG LCKNLLQEFAQKHGYPLPQYKSVRQGEEHSLVFSSTVEIAGVSYSGGCAKSK KEAEIKAARTALLAIQATQPAEPHIAVSPVDQPGLVANFVKAQQKRKRKGRL HPVVVEEPLATRVKQCVGQIPEPVQLNQVHQVPYMHQIPQVHQMSQGPQVP QIPQYHHVIPVNQVGQQYSVNPGTFPAQNVSPNDVKKEPMSNVGAHDNLVT QLDIGKKSVASHKPVAGTESKLVAKVVDVKEAAVSDVQRAAVEVEPTAVHQ ISITTDVIKESRNVKISNVKANAVLETLTTGSKRNSPLIEPRVNEESSPSQKLVP TALKSPAASQRAVGRNFMDVQLPIKRRHKDH*

>DRB1_Prunus_persica[ppa004305m.g]

MATNEGFQGVSNCYVFKSQLQEYAQKVEIPTPVYETIKEGPSHEPSFK STVIVNNVRYDSLLGFSNRKLAEQSAAEVALVELAKSGDINQSISQPIHETGLC KNLLQEYAQKMNYAIPLYQCQKEETPGRVPLFSCTVEIGGIRYVGAAAKTKK EAEIKVARTALLAIRSSKSESSMEPVGNTQLTVLPSKKRRAESNTKSEETANVP

KPKKGRFKKRNFKKKLSGDMVGQTQVRNVGDMEVNTFSAKKGESTASFFN
HGDYGTLDEGAFPISYGNITPLAKEVNMVSGAAEVVSLANNSNLQLFNHGDD
NGTLDVEAYPISCGNLTSVAEEENMVSGAGESISLANNSNLQLFNHGEDNGT
LDVDPISCGNITSVAEEANMVSKVGEAVSLANNSSLQLFNHSDDNGTHDVGA
YPISCGNITSVAEEANMVSGAGEVVSVADNSNLQLFNHNDNRTLDVGANPIS
CGNVTSVAEEANMVTKAGEAVSLANNSDIHYLEASSVMAGLNQSGNESKQV
*

>DRB1_Ricinus_communis[29908.t000326]

MCTLPAMCSSVSNCYVFKSRLQEYAQKKGLPTPVYETIKEGPSHEPSF
RSTVIVNDIRYDSLPGFFNRKAAEQSAAEVALMELAKCDEVNDSISQPVHETG
LCKNLLQEYAQKMNYAIPLYLCQKNESPGRGTLFKCTVEIGGIHYIGASAKTK
KEAEIKAARTALLAIQLSASESSHNSIGNCQLTVIPFRKRGAETAAVLEEAMN
VPKAKKARFRKKTLKKKHSGNKVDHNQGETIGNKKDGQAGSESDKNDASV
VQGTGHQLLPMLSTSNSEDGRLVPESSERETTDVTGALVCHISGDFGNGFAAT
VNFDQSNHGVCAETNGDTAKFSGATLVQIGAATVMAGLNPAGERIEAGAGQ
*

>DRB1_Selaginella_moellendorffii[SELMODRAFT_106185]

MYKSQLQEFAQKSGWTVPQYDSIKQGLPHLPRFQASVEVNGVKYESE
DGFPNLKAAEHSAAKKALDSLTGGANGASTDASGSSMTGLCKNVLQEYAQR
NGFSLPIYQIEITGPSHNSVFAATVEIGGVLYKGGTAKSKKEAEVKAARTAILA
IKELAGKECFSFLVSLISSLSFFSFSLRGRSFRRLRSTTVWKKASESCQEQDRER
ITTTQTTGVRDLEAAKLVTQTSILCLCICSDYLDEELELFVFRASTRSDENLFLR
ISSSLSSFWSSFVAAVSRASSTNGSFTTPFQAFFDPV*

>DRB1_Setaria_italica_[Si010100m.g]

MDGGGAAPHDAATNISPAPNVAAAPTGIRVENCYVFKSRLQEYAQKA

GLPTPEYHTLKEGPSHEPVFKSTVVVNNTKYDSLPGFFSRKAAEQSAAEVAL
MEIVKSVPPTETKSIPAVQETGLCKNLLQEYAQKMNYAIPSYICTKQASGVAP
FVCTVEIGGIQYIGAAARTKKEAEIKAARTALLAIQGQSEGCGNGATKYIVVP
GQREVKETDKKPTETPKSLKVKKSGGRKKWNKRKFMRKTDQIVDAEKDGA
REAGDVHDSDVPMQTTITEEPSRDSIILHLDDEARRVEMEHLRDVATLQPDKE
ARSVNQGLAMLLHSEEAIRVEHDLPRDTAMVQSNREVVMLQSDEEARKPPR
DPVTVQPNEEARSVKQEPLSSAEAAKPNMEGRTVEEESARAYVALQFNRDA
KDVKEELPSNTVMMQCEETETIKQEAPQSGELEEPPN*

>DRB1_Solanum_tuberosum[PGSC0003DMG400009356]

MANNESIRSVTSCYVFKSRLQEYAQKVGLPTPVYETIKEGPSHEPTFTS
TVIVDNVRYDSLPGFYNRKAAEQSAAEVALMSLGNTGSMENLSQPVHETGL
CKNLLQEYAQKMNFAIPQYECERYDSESKIISFSCTVDVGGMKYIGAAARTK
KEAEIKAARTALLAVQSSGFAPNYSSYTVVPMKKVTDLAISNQESAAALKPK
KNRFKKKQTRNVSDASYRVRTKNTGDSEVQMVNQAKPEMHENAAVMTQG
TGSGPAPFAGTMGDLVPSNNLSSDYGTSNLGINSQCGGGVTTEGNAVIGVEQ
VTSELAPVAWNDNLHVQDLPLLHENAAVMTQGTGSGPAPFADTMGDLVPSN
NCGSDFGTSNLGINSQCGGGVTTEGNAGIGVEQVTSEVAPVACNDNLHVL*

>DRB1_Sorghum_bicolor[Sb08g000900]

MDGGGAASPDTAICIAPGPGPIVAVPPAGIRVENCYVFKSRLQEYAQK
AGLPTPEYHTLKEGPSHEPVFKSTVLVNNTTYESLPGFFSRKAAEQSAAEVAL
MEIAMSAPVAETRSIPAVQETGLCKNLLQEYAQKMNYAIPSYICTKQASGVA
PFICTVEIGGIQYIGAAARTKKEAEIKAARTALLAIQGQSEGCANGAKKYIVVP
GQRPVKETDKKPTETPKPLKVKKRGSKRKWNKKKLVGMANQIVDAQKDVE
GLGMLLQYDHEEAGRIEYELSRDTAMVQFNKEVIVMQPGEGDRIVQPESPRD

PAQVQHNNAARSVEQDPLSNTEVVMPNNEAITIEHEPLSAYVPLQSNGDSTD
VKEAPSNTSMMQGEESETTRQEAPHTGELVQPN*

>DRB1_Theobroma_cacao[Thecc1EG034521]

MPTNENFSGVSNCYVFKSRLQEYAQKVGLPTPVYETIKEGPSHEPSFRS
AVIVNDVRYDSLPGFFNRKAAEQSAAEVALMELSKSGEVNQSISQPVHETGL
CKNLLQEYAQKMNYAIPVYQCLKDEAPGRGPHFSCTVEIGGIRYIGAAARTK
KEAEIKAARTALLAIQSSTLELSNKVVGNSQLTVIPCRKRAMETASNPEEAVN
VPKAKKTRFKKKMLKAKLSGNSVDHSQDKSTGNSAVGMDDPVKSEWVQTN
SLSSETLATEVVGNLQDTKLDSDLIEREVPSAEVALPPQGADNSKNGQLTALN
CVHCNHEAPDVGNSSMVYADVTALVKVTDGVEVASMVNDSSFSQMEASKI
MTGLNQAVERIHANAGQA*

>DRB1_Vitis_vinifera[GSVIVG01009189001]

MSSNDGFAGVSNCYVFKSRLQEYAQKVGLPTPLYDTIKEGPPHVPSFK
STVIVNDVRYDSLPGFSNRKAAEQSAAEVALVELAKSGNMKECISQPVHETG
LCKNLLQEYAQKMNYAIPMYVCQKDESPGRAASFSCTVEIGGIRYIGAAART
KKEAEIKAARTALLAIRANTDGVGEKSIGNTQLTVIPGKKKGPESGNPDEPAK
ALKPKKARFKTKGLRKKFPGNKVGNDQVEDTRGMEVDIDNQVGSELLQTDA
SMVQVAESGLLSMESKENFEEEKSIVNQNGGDKTVAEGDPIPDIIGNCQNGQA
TASDIKKTDHKAPPELGISSMSPAEVLVSVAMEVNNVSEAGLVDSAIMNSSNE
DQTEAASAAKDFKQMGANEDDFKSNGASALGIVASNLGDTLSLFLFELSPSY
L*

>DRB1_Zea_mays[GRMZM2G179031]

MYKNQLQELAQRSCFSLPSYVCTREGPDHAPRFRAAVTFNGETFEGPS
GCTTLRQAEHAAAEVALARLSLRGPSTTLAARVLDETGVYKNLLQETAHRA
GLKLPAYTTVRSGPGHSPVFSSTVELAGLSFAGDPARTKKQAEKNAAMAAW

SSLKQSTSSAQSRKHKPCAYLYASRGIINGPRHGLCSAGGSQGARRRRRAGA

RGCRQSSRGAEAARRRRRKGGAASAEAQQQRLFFLRSSEPVAVQTPMAASD

QYPCPSPTPAAEDGGPAPAAAGRPKDPAPTASATTAGVRLQGRRGGRAGAD

AGARHGARPGGNAAFAVLLRGACRLGVPPRQRAEDLRRGRVPRPGCERAVG

DPGVRRPAAADPGQGGKEWPGDTLRRMQEGVSDVVSL*

DRB2

>DRB2_Arabidopsis_thaliana[AT2G28380]

MYKNQLQELAQRSCFNLPSYTCIREGPDHAPRFKATVNFNGEIFESPQ

YCSTLRQAEHSAAEVALNALSNRGPSHSLAARILDETGVYKNLLQEIAQRVG

APLPRYTTFRSGLGHQPVFTGTVELAGITFTGDPAKNKKQAEKNAAMAAWSS

LKQLAKETSSSMPEPENIDELEQVIIARALINYRIKENIGTGSSSSAPVPFAKKFF

MQNLRPTSPQPSPATTSRILPFICPKQPSRSSRSSLAATSGIDRIMAAALESRSY

QRPQQRFANPPYVPMRQFRSQCHGMAPPVTIRTAVPVFSAPPMPPPPCTNNTQ

LPSSVYVPSLMRTAPPVRIAPPVTIRTAVPVFASAPPVRIRTAVKPTVEAGETRI

SSVQEKESIPVLPDSLEIGVEGSTITITDCEKTASKETERAEFKDSSKGEPETAR

ERLENLKI*

>DRB2_Aquilegia_coerulea[Aquca_009_00003]

MYKNQLQELAQRSCFNLPWYTCIREGPDHAPRFKATVNFNGEVFESP

HFCTTLRQAEHSAAEVALNSLSNTNRGPSHSLASRIIDETGVYKNLLQEVSQR

VGAALPQYTTLRSGLGHFPVFTCTVDLAGITFTGHPAKNKKQAEKNAAMAA

WASLKQLVKQDPSSSSEAEHSDEQEQVTIARALLNYRLKEKMAMAESPNALS

PFPKKFPIQHQRPSGTQPPSITTSKILPLIRPKSDARIRPIVMVQNDKRQPPQFPV

PENSGKRPQKFPALDAIPYVPIRHFRAPYHGVAPPVTIRTTVPVFSAPQLRPPT

NRASLVMASPTVQISPPVHTRETLPVFSAPLPKLEEPMVVTISPTTNPSIPIEVIG

KIEVSAVPQVCIKEPPIFPVPPPKPLTHRDEIGNKTAKDLQKSVIPGSVVSSLPTS
SLPTSSLGTFSLAAKSPLHSQDVENKTGDDLEEYAMVQGVKQLRI*

>DRB2_Amborella trichopoda[AMTR_s00148p00030810]

MYKNQLQELAQRSCFNLPSYSCIREGPDHAPRFKAAVNFNGEVF
ESPGFCTTLRQAEHAAAEVALNTLSNRGPSQSLAARILDETGVYKNLLQEIAQ
RAGAALPVYTTVRSGLGHLPVFTCTVELAGISFSGEPAKSKKQAEKNAAMAA
WSSLKKLAQQQQAASSFTSSLSSPTSSEGATPSPEPSDEQEQITIARALVNFRH
QLLNNSNKSNPCSPSLPGAKLSFPNQHNPKPFTQNLGSKILPLIRQRQTQRPNP
HAPPFIPPENRILATQAQVPASQSGFPATQNSFPAGQNRISAIQTQIPAGVGPVF
YPVGPAYRPTVHHAAPPVTIRNAIPVFSARPSHVRMPPPVQVRHVAPVFTTGP
MATAPKVGERAERVDDKVLERGLKDLQI*

>DRB2_Brassica_rapa[Bra035704]

MYKNQLQELAQRSCFGLPSYTCLREGPDHAPRFKATVNFNGEIFESPQ
YCSTLRQAEHSAAEVALGALSNRGPSHSLAARILDETGVYKNLLQEIAQRVG
APLPRYTTFRSGLGHQPVFTGTVELAGITFTGDPAKNKKQAEKNAAMAAWSS
LKQLAKETSNSMPESENSDELEQVIIARALINYRIKENIGSGSSSSAPVPFAKKF
FMQSLRPTSPQPSHATTSRILPFICPKPSSRTSRSVSAASVERAIAAALENRNYR
PPQQRFAAATPGAAAAPPYVPMRHMRSPCNGMAPPVTIRNSVPVFSAPPPTC
ANTQQQQRPSVYVPSMMRTAAPVRIAPPVTIRTAVPVFASAPPQIRKEEALPV
RKVNVQTSVKPVVQEREERALSLPDNLEIELEASAKPVSETDRAALKDSKGE
HETVKERLENLKIW*

>DRB2_Brachypodium_distachyon[Bradi3g28977]

MYKNQLQELAQRSCFNLPAYTCLREGPDHAPRFKASVTFNGELFESPG
FFTTLRQAEHAAAEVALAALARRGPSYSLAARILDETGVYKNLLQEVAQRVG
APLPSYTTERSGLGHLPVFTCTVELAGIIFTGDHAKNKKQAEKNAASAAWAS

LKQLAREEANSTNEPENNDEQEQIRIARALLNYRLKEKMAMANNPHASPFPK
KFPMKPERKPSFAQSSQSSYSKILPLFRPKSSSRSRPESPAATDGASQSPFWSIE
TSNSRSRFPAAGAAPYVPVGHYRMPCHSMAPPVTVRSAVPVFSAPPLPPPGSQ
TQQLPPLLSHPPPIRMASPVRIRPPPALFAPSGPVRSPRPVMSVQMKDVQHKP
MKESLSSVIPLQVKDAQHQLFNGSLSPVIPIQMKDVQPQLPKEQLSLAKDAPP
VVPLPVIRPPVKIEAPAQVKEAPQVVTSEVQCPAAGSTAAATSAEFLPASQSG
AADEDKGEAALDDQAEAKAAVEGIIRHLE*
IK*
>DRB2_Capsella_rubella[Carubv10023224m.g]
MYKNQLQELAQRSCFCLPSYTCIREGPDHAPRFKATVNFNGEIFESPQ
YCSTLRQAEHSAAEVALNALSNRGPSHSLAARILDETGVYKNLLQEIAQRVG
APLPRYTTFRSGLGHQPVFTGTVELAGITFTGDPAKNKKQAEKNAAMAAWSS
LKQLAKETSSSMPEPENIDELEQVIIARALINYRIKENIGTGSSSTAPVPFAKKF
YLHNPRPTSPQPSPATTSRILPFICPKQPSRSSRSLATPAGVDRIMAAALESRSY
QRPQQRYANPGTAAPPPYVPMRHLRSPCHGMAPPVTIRTAVPVFSAPPMPPPP
CTNNTQLPSSAYVPSLMRTAPPVRIAPPVTIRTSVPVFASAPPVRVRKVDIQTA
VKPAVEVGETRISLVQEKESVPVLPDTLEIGEEAIYIPISGSEKTPAKETERADL
KDLKGEPEIARERLENLRI*
>DRB2_Carica_papaya[evm.TU.supercontig_216.1]
MYKNQLQELAQRSCFNLPSYTCIREGPDHAPRFKATVNFNGEIFESPH
YCSTLRQAEHSAAEVALNSLSNRGPSHSLAARILDETGVYKNLLQEIAQRVG
APLPQYTTFRSGLGHLPVFSGTVELAGITFTGDPAKNKKQAEKNAAMAAWSS
LKQLAKETASTSSEPENNDELEQVTIARALLNYRLKEKIAMANSPNAVIPFTK
KFPVQTQRPTSPQPPPATTSRILPFICAKSSPRNRVGSMSVNDRSMPASQATAL
EVRGTRPQKFPAAGAAPYVPMRQYRTSCHGIAPPVTIRTAVPVFSAPPLAPPS

VLSCQPPANANQPQPSLSLLSPQPAPASTHPSQPPSAVLPTQPPACSQSPTSMIS
SQPPASVNLPKPPTSLLAQQPPPSALPSQLMRTSPIRIAPPVTIRQAVPVFAAPP
VQKESLAVRKEDNMGVIFPAMPNKSTAHVEETGATAAAKSLQVSQTVQSLK
QLKI*

>DRB2_Citrus_sinensis[orange1.1g012154m.g]

MYKNQLQELAQRSCFNLPSYTCIREGPDHAPRFKATVNFNGEIFESPH
YCSTLRQAEHSAAEVALSSLSHRGPSPSLAARILDETGVYKNLLQEIAQRVGA
PLPQYTTIRSGLGHLPVFTGIVELAGIAFTGEPAKNKKQAEKNAAMAAWTSL
KQLAKETASSSSEPETNDELEQITIARALLNYRLKEKMAMASTLDSPIPFARKF
PVQNTRPTSPQPPLATTSKILPLFCPKTASRHRPASSGANEKPAQPQPYGSEGR
VVRPQKFPAAGAAPYVPIRQYRTSCRGIAPPVTVRTTVPCFSAPPHPPPSALPP
QMMRAPAVRIAPSVTVRQAVPVYAAPPVHRDDSLTVRKEDPPTTPAPAQKV
DSWTVFPPAILKDLPTVTAAAIQKEDTPTVTPAIQKQDCLTVASPVVQKENPPI
VVASAVQKGYPPNSTAPSPQEILSQVEKRENTILNIEETEAACSLGQLKI*

>DRB2_Cucumis_sativus[Cucsa.200460]

MYKNQLQELAQRSCFNLPSYTCIREGPDHAPRFKATVNFNGEIFECPQ
YCSTLRQAEHSAAEVALNALSNRGPPHSLAARILDETGVYKNLLQEIAQRVG
APLPQYTTFRSGLGHLPVFTGIVELAGITFTGEPAKNKKQAEKNAAMAAWSA
LKQLAKESASSSSEPENNDELEQITIARALLNYRQKEKLAMSNPNATIPFHKKL
QIQTPRPTSPQRPPAPTSKILPLICQKAAPRSRAPFSANKIPIPQSQTPALEGSGP
RPQKFSAGTALSYIPVQQFRTSCHGIAPPVTIRTAMPVFSAPPLPQPSKLPPQQV
IRVPPIRIAPPVSIRQAIPVFAAPPVRKENPPV*

>DRB2_Eucalyptus_grandis[Eucgr.J00927]

SSLSLGPEEDRTRLLPPYDRREQRKTLGRKPPDLRRAFAPPFAGTGTGA
LIRSRPDALEPGSELVSGRGIDSTPRRSGGGGGGGGEEGGGDMYKNQLQELA

QRSCFNLPSYACIREGPDHAPRFKAVVNFNGESFESPSYCSTLRQAEHSAAEV
ALHSLATRGPSHSLASRILDETGVYKNLLQEIAQRVGAPLPHYSTFRSGLGHL
PVFTGTVELAGIIFTGVPAKTKKQAEKNAAMAAWSSLKQLSKETASTSSEPEN
NDELEQITIARALQNYRLKEKVALANSPSGTIPFAKKFQIQI*

>DRB2_Fragaria_vesca[gene21565-v1.0-hybrid]

MSILLFACVSIGLYVRRRSELIIGDDGNMLGFDVRPGAVAFPLQANWV
VVGHVPGRKCADADVGPLKGVDCGDLIGHVLKGTGRLIGARVPNPPERKVD
VLICVELAFYIKMRFVVKPQEQIREGRWAQCGQYLNVILAILHVSVPTVTDVR
SSPPLGRCLMSASSSSLLDIPYGEIYLIADLSFLSLQNPTHFPPESSGPTLFAADPI
WRVEGEMYKNQLQELAQRSCFNLPSYTCIREGPDHAPRFKATVNFNGEIFESP
HYCNTLRQAEHSAAEVALNYLSNRGPSHSLAARILDETGVYKNLLQEIAQRV
GAPLPQYTTFRSGLGHLPVFTGTVELAGITFTGDSAKNKKQAEKNAAMAAW
SSLKQLAKETASSSSEHENNDELEQITIARALLNYRLKEKMSMANPSGPILFPK
KFPAQNPRPTSPQPPSAMTSKILPLICQKQAPRSRFQSNASNATNESLVPSSHSS
PVESRWTRPQRFPAAGAVPYVPIRQMRTPCHRMAPPVTIRTSVPVFSAPPLPPP
PGLHRQTMCVPPIRVAPPVTMRQAVPVFAAPPVRRDDSKKDDLPTSRKEESPI
VPKEAAPAVIAPAVTAAASPAVIAPPLPNKSIAQVVEKSGSTSANDLQESKTV
QGLEQLKI*

>DRB2_Glycine_max[Glyma12g08070]

MYKNQLQELAQRSCFNLPSYTCIREGPDHAPRFKATVNFNGEIFESPH
YCSTLRQAEHSAAEVALNSLSNRAPSHSLAARILDETGVYKNLLQEIAQRVG
APLPQYFTFRSGLGHLPVFTGTVELAGIMFTGEPAKNKKQAEKNAAMAAWS
SLKQLAKETARSSTEPENNDELEQITIARALLNYRLKEKISMSNSNAPVPFPKK
FQIQNPRPTSPQPPPAATSKILPLICQKAAPRSRHLVGASPARASCDNSAMPQL
SATPESRGIRRPKFPAAGAAPYVPIRQMRPCQGMAPPVTIRTAIPVFSPPPAAA

AVSHQVLRAPHVRVAPPVTIRQAVPVFATPPPPIRKDEPVPIPKDEPPTISAPSP EDKLPAKTPEAETKTENIPPKPDMVKSLEQLKI*

>DRB2_Gossypium_raimondii[Gorai.003G142000]

MYKNQLQELAQRSCFNLPSYTCIREGPDHAPRFKATVNFNGETFESPH YCSTLRQAEHSAAEVALQSLSNRGPSHSLAARILDETGVYKNLLQEIAQRVG APLPQYTTFRSGLGHLPIFTGTVELAGIRFTGEPAKSKKQAEKNAAMAAWMS LKLLAKETASSSSEPENNDELEQITIARALLNYRIKEKMAMANSSNAPILFTKK FPSQNPRPTSPQPPATTSKILPLICPKVVPRNRSMSATANEKPVQTSSQTPTPES RGVRPQKFPAAGAAPYVPIRQLRTPCCGIAPPVTIRTAVPVFSAPPRPAPSAVS PQPPTSLVPTHPAQPAQSVLPPHQLPATLPSQVLRAPPVRIAQAVTIRQVVPVF AAPPVRKEDKQSVPLRNEDITTATAAPPPNQSPTQAEEAASTILKNLQESETV QSLEQLKI*

>DRB2_Linum_usitatissimum[Lus10021471.g]

MYKNQLQELAQRSCFNLPAYTSIREGPDHAPRFKATVNFNGEVFECPH YCTTLRQAEHSAAEVALNSLSHRGPSHSLAARILDETGVYKNLLQENKNLLQ EIAQRVGAPLPQYTTFRSGLGHQPVFTGIVELAGISFTGDAAKNKKQAEKNAA MAAWSSLKQLAKEESSSSSEPENTDELEQITIARALLNYRKKERMAQANSPN APIPFQMKFPPQNTRPTSPQPAPAATSRILPLIYPKPTRNRPMVAPDRHQYSRS TSATPNDRFAPPRPTNDRHVPARQPNVLDLWAHQPQPYIPMQPFGQTCRSTV QPVTIRHAVPCYAAPPQRPMVVPSHSVRAPLIGRNVMPVTIRQAVPVFAAPPP SVHKVDSPTMQKDSIESKKDLSVEFKQQESKKDYPVEFKQEEPKKEESKDSM ESKQEGAIPVAVIPAQVEEEKQQIANCTTEVKESSKVEASLEKLKI*

>DRB2_Malus_domestica[MDP0000188414]

MYKNQLQELAQRSCFNLPSYTCIREGPDHAPRFKATVNFNGEIFESPH YCTTLRQAEHSAAEVALNYLSNRGPSHSLAARILGFANVRRDETGVYKNLLQ

EIAQRVGAPLPQYTTFRSGLGHLPVFTGVVELAGITFTGEPAKNKKQAEKNA
AMAAWSSLKQLAKETASSSSEPENNDELEQITIARALLNYRIKEKMTMANPT
APILFPKKFPVQNRRPTSPQPPPAHTSKILPLICQKQAPRSRYQSNATHDNRVT
SSQASPLDSRVMRPQRFPAAGAAPYVPIRQLRTPCHGIAPPVTIRTAVPVFSAP
PLPPPHAMPCQTMRIPPLRVAPPVTMRQAVPVFSAPPVKKDDPPTTRKEESPVI
VKEDPXIIVKEDSXIILKEDPPIIPKDPAAVVAPCVQNKPIGQVEETVRXAADD
LEESKTVKILEQLEI*

>DRB2_Medicago_truncatula[Medtr2g094490]

MYKNQLQELAQRSCFNLPSYTCIREGPDHAPRFKATVNFNGEIFESPH
YCSTLRQAEHSAAEVALNSLSHRGPSHSLAAKILDETGVYKNLLQEIAQRVG
APLPQYTTYRSGLGHLPVFTGIVELAGITFTGEPAKNKKQAEKNAAMAAWSS
LKQLAKETASSSTEPENNDELEQITIARALLNYRLKEKMSMSNPNARIPFQKK
FQIQNIRPTSSQSPATTSKILPLICQKTGPRNRPSSATPNENPRSRHPQAAATSD
KSILQQPQSSAIESRVTRPLRFPAAGAPPYVPIRQMRPCHGIAPAVSVRTVIPVF
SAPPLPPPTSVHQIIRAPPVRIAPPVSIRQAVPVFAAPPLRKDEPAPIQKDLPASS
TPVEQDKLPAKILEMDKTENNSPPQPETLQSLEQLKI*

>DRB2_Mimulus_guttatus[mgv1a008321m.g]

MYKNQLQELAQRSCFNLPSYTCIREGPDHAPRFKAIVCFNGENFECPH
YCSNLRQAEHSAAEAALASISSRGPSQYSLAARILDETGVYKNLLQEIAQRVG
SPLPRYTTFRSGLGHLPVFTGTVELAGIVFKGEPAKNKKQAEKNAALASWLS
LKQLAQQDASSSTEQENNEEQEQIRIARALQNYRQKEKSETGNLNGVPIPFQQ
KFSVPTARPSISTSKILPLFCQKNASSRNSRPSSIINDGHTTQMFTAFGAAPYLP
HTSHCGLPYQGIAPPVTMRTSIPVFSAPPSGPPSVSIRPQRPMGIAPPVSIRPQRP
TCIAPPVCIRQAVPVFAAPAGRQDPPPSKLLYKTDEAEKNESNCEVDESTAIRC
LEQLEM*

>DRB2_Oryza_sativa[LOC_Os10g33970]

MYKNQLQELAQRSCFNLPAYTCLREGPDHAPRFKAAVNFNGEQFESP GFFTTLRQAEHAAAEVALAALARRGPSYSLAARILDETGVYKNLLQEVAQRV GAPLPSYTTERSGLGHLPVFTCTVELAGITFTGDPAKNKKQAEKNAASAAWS SLRQLVRQEASSSNEPESNDEQEQIRIARALLNYRLKEKMAMANNPHASPFPK KFPMQPERRTAFPQSSHSSYSKILPLFRPKSNSRSRPESPAASDAASQTPFRPTE SPNPRSRFPAAEAAPYVPVGHFRMPCHSMAPPVTVRTSIPVFSAPPLPPPGART QQLPPLMSHPPPIRMASPVRIRPAPPLFTPSAVQGPKPMMPVQIKDVQHQQIKE TRSPVMPVQVKDAQNQLLKGSLSPVIPVQIKDVQSQPPKEALSPAIPVQIKDV QLQPRNEPVSIGKGVVPLPAIRPPVKVEAPAEVKEASQPVAGSSVVQCKADTS PDSLPKTQLKTANADNADAKDDHLPVDAEEVEDIIRHLELK*

>DRB2_Phaseolus_vulgaris[Phvul.011G079700]

MYKNQLQELAQRSCFNLPSYTCIREGPDHAPRFKATVNFNGEIFESPQ YCSTLRQAEHSAAEVALNSLSNRAPSHSLAARILDETGVYKNLLQEIAQRVG APLPQYFTFRSGLGHLPVFTGTVELAGIMFTGEPAKNKKQAEKNAAMAAWS SLKQLAKETARSSTEPENNDELEQITIARALRTYRLKEKISMSNPNAPIPFPKKF QIQNPRPTSPQPPPAATSKILPLICQKAASRSRHPVAASPAAAVSDNSAMPQLF ATSDSRRIRRPKFPAAGAAPYVPIRQMRSPCHGMAPPVTIRTAIPVFSPPPPTA ATLSLPVLRAPPVRVAPPVTIRQAVPVFAAPPVQIDEPVPSLTSLEPVTTPKDD APTISSPSQEEKLPVKIPEIEIKTEKIPAESETVQRLMQLKI*

>DRB2_Physcomitrella_patens[PHYPADRAFT_88725]

MLSLSLSLSLSLSTLALSPAIDGVDVSAYVVKAAPHSGAVSFPQSLGPL KRWTVGVVREWAFDCMYKNQLQELAQRSCFNLPAYACIREGPDHAPRFKAT VNFNGEVFESPNYCNTLRQAEHAAAEVALNTLSRRGPSQSLAARILDETGVC KNLLQETAQRAGVSLPVYATTRSGPGHLPVFTCTVEVANMSFSGEAAKTKK

QAEKNAAMAAWSAIQQLANQGRGVPLATEGEVSEEQEQNTIARALAQHYG
KESQQLPHSTQNPSSSVMPIRLRTLTSRDGLQPGSPRLNQSHSGPWATDLSME
QQRHTRNQGHSSVGPVASPAYRPVGTSRIGSSVTIRDVTGHRDSAALRDASA
MRDAALAKRAVERAMCGNFVGRHSPVNLRPVPQMRRDRHEVAVHYDNHQ
RDEDEWLRGESTKASRDVDCLNDSLEELGASAVHGSYNPMSWSGAGVSNW
WNMHPAAMPRASSRAAMPVVLRPAVMVCAAPPLRPEPDDAENEGEAATHQ
GMLTRSSGDVSVRVHRMDSCLQVSLSVILTVYGSCKRDGECGGIFVSGASCG
AISHVYSNGIVLIGDFVLTEGRQPRKGNSNIQLLQALAGRIGNASQVQLGCDD
DDCVRVKRELYFRTKLWHTIVDQLVTAGIGISSRVGVAGHRDAELCAAWQN
PREVHICVIAWKQLSFVFAV*

>DRB2_Prunus_persica[ppa024708m.g]

MLHIPQLQSPKPLSLSNSISHPHPKKTLKTSDLSRSHFSPAPQNPNPAST
RPEIGADPIRAPPIIAPELGAPRRVGWETESERERQDRVRGGEMYKNQLQELA
QRSCFNLPSYTCIREGPDHAPRFKATVNFNGEIFESPQYCSTLRQAEHSAAEVA
LNYLSNRGPSHSLAARILDETGVYKNLLQEIAQRVGAPLPQYTTFRSGLGHLP
VFTGTVELAGITFTGEPAKNKKQAEKNAAMAAWSSLKQLAKETASSSSEPEN
NDELEQITIARALLTYRLKEKMANPTAPILFPKKFPVHNPRPTSPQPPSAHTSKI
LPLICQKQAPRSRLQSNATNDNRIPSSQPSPLESRVLRPHRFPAAGAAPYVPIR
QLRTPCHGIAPPVTIRTAVPVFSAPPLPPPPVMPCQAMRVPPLRVAPPVTMRQ
AVPVFSAPPIRKDDPPTTRKEDPPIIVKEDLPVIRKADPPA*

>DRB2_Ricinus_communis[27538.t000020]

MYKNQLQELAQRSCFNLPSYTCIREGPDHAPRFKATVNFNGEIFECPH
YCSTLRQAEHSAAEVALTSLSNRGPSHSLAARILDETGVYKNLLQEIAQRVGA
PLPQYTTFRSGLGHQPVFTGTVELAGITFTGEPAKNKKQAEKNAAMAAWSSL
KQLAKEDASSSSEPENSDELEQITIARALLNYRLKEKMAIANSPNSPIPFSKKFP

MQGPRPTSPQPVPATTSKILPLFCPKTTRNRPTSTTITDRSVPTRTATSTTSCDR
TVPPRQSPTLDFGSSSSEIPAAEQLLCPYPTIGRMPWHGSPVRVRSVVPVFAAP
QCQPPSLPPQVLRGLPQQPPPVTIRQTSLVYAASPPVQKEDPLNVQKGNVVVP
KEDSPASQKEHPDIGIEDSPTVKKEHSAVQKADSQAGQTDNPAVEVEDPLCN
TKEDQAAENPNKSPAQLEEASTSTNKSLQESAMLQSLEHLKI*

>DRB2_Selaginella_moellendorffii[SELMODRAFT_431451]

MYKNQLQELAQRSCFNLPAYSCIREGPDHAPRFKAAVNFNGEVFESPN
YCSTLRQAEHAAAELALNVLSRRGPSQSLAARILDETGVFKNLLQETAQRAN
VPLPTYTTTRSGPGHLPVFTCVVEVAGMNFTGDAGKTKKQAEKNAAMAAW
ATLKQFAKKLAPPSLFYSDEMTEDQEQISIARVLYLAYEKVGGGQASRPRQY
TVPSLFDKDCTVGYLGSQQQHLRSGAGFCSTNPRSSNPEALALPRDIRAYGM
THPRVRTRNVQLTHEVPPPIEEHRRDEEDWLRGESSSSSSTCASSSRPMPEEPK
RSSRSPMFWPRTTDSHWWQSDSSLLSSGYHLRRPQVSLAPPVRVRSVVAVSA
APPQRSQPQEAADQDGLSDGLSRLNL*

>DRB2_Setaria_italica[Si034815m.g]

MSRLCLLALGPSCHKPAKVTGVGPQLPSRSTRTTSLFFPSSPLLLVDLS
GLAVSGHRGRHSEERRGSGRRSAAMYKNQLQELAQRSCFNLPAYTCLREGP
DHAPRFKAAVNFNGEQFESPGFFTTLRQAEHAAAEVALAALARRGPSYSLAA
RILDETGVYKNLLQEVAQRVGAPLPSYTTERSGLGHLPVFTCTVELAGITFTG
DPAKNKKQAEKNAASAAWSALKQLVREEANSSNEPENNDEQEQIRIARALL
NYRLKEKMAMANYPHASPFPKKFPMQPERKPSFGQSSQSSYSKILPLFRPKSN
SRCRPESPASTDGVSQTALRTLESLNPKSRFPAAEAAPYVPVGHYRMPCHSM
APSVTIRTAVPVFSAPPLPPPAARTQQQLPPLMSHPPPIRMASPVRIRPASPMFG
PSAPVQGPKPVMSVQLKDVQQQSRREPVKPVIPVQVKDVQYQPMKGSVSPV
VPVQVKDAQRRPLVGSLSGVIPIKVKDVQTQAPKESLAAPIPAIRPSVKIEAPA

QAKEASAAVTSEVPCSAAGNTTAVECTTSSEVTPTRQSRAADGDDSKAEAVH
EAEAQAVAEAAIRQLEIN*

>DRB2_Solanum_tuberosum[PGSC0003DMG400008053]

MYKNQLQELAQRSCFNLPSYVCIREGPDHAPRFKAVVNFNGESFESPH
YCSTLRQAEHAAAEVALNALSNRGPSHSLAARILDETGVYKNLLQEIAQRVG
APLPQYTTYRSGLGHQPVFTGTVELAGITFTGEPAKNKKQAEKNAALAAWSS
LKQLAQQDASLSSELENNDEQEQIRIARALLNYRLKEQLEMAKSGKVPIPFQK
KFPMPSPRPSSPQRPAVTTSKILPLICPKTVNRYRSSSTTINDSHSSLSQPLPQSQ
TSSSEGRTVSTRISPAPYMPVRQYTRTPYHGIAPPVTIRTAVPVFSAPPRPQPTG
CPAQMMQARPVRVAPPVCIRQAIPVYAAPPVKKETVAHATTTLSRPSAQPEE
TVARATTAPSRPLAQPKETGTNAGTEVDESTAMKCLEELSL*

>DRB2_Sorghum_bicolor[Sb01g019110]

MYKNQLQELAQRSCFNLPAYTCLREGPDHAPRFKAAVNFNGEQFESP
GFFTTLRQAEHAAAEVALAALARRGPSYSLAARILDETGVYKNLLQEVAQRV
GAPLPLYTTERSGLGHLPVFTCTVELAGITFTGDPAKNKKQAEKNAASAAWS
ALKQLVREEVNSSNEPENNDEQEQIRIARALLNYRLKEKMAMANYPHVSPFP
KKFPMQPERKPSFGQSSQSSYSKILPLFRPKSNSRSRPESPASTDGVSQIPSRAM
DSLTPSPRSRFPAAEAAPYVPVGHYRMPCHSMAPSVTIRTAVPVFSAPPLPSPS
ARPQQLPPLMSHPPPIRMASPVRMRPSSPMFASSAAVQGPKPVMPIQLKNVQ
DQSRKETAPSAIPVQVKDVQYQPRKSSMSPVIPVSVKDAQRQPLPVQMKDVQ
TQAPKESLSAPIPAIRPSLVKIDLPAQGKEASASATSEVPSSATGNNAAVECTA
SSDALLARQSRAADGDKDKAEAKHEAEAQAVAEAAIRQLEIN*

>DRB2_Theobroma_cacao[Thecc1EG042059]

MYKNQLQELAQRSCFNLPSYTCIREGPDHAPRFKATVNFNGETFESPH
YCSTLRQAEHSAAEVALQSLSNRGPSHSLAARILDETGVYKNLLQEIAQRVG

APLPQYTTFRSGLGHLPVFTGTVELAGITFTGEPAKNKKQAEKNAAMAAWTS

LKQLAKETASSSSEPENNDELEQITIARALLNYRIKEKMAMANSSSAPIPFTKR

FPIQNPRPTSPQPPATTSKILPLICPKVVPRNRSMSATANDKHILPQSQASIPESR

GTRPQKFPAAGAAPYVPIRQFRTPCHGIAPPVTIRTAVPVFSAPPRPAPSAVSS

QPPASAVPTQPAQSVLPAQPPQSAVPRLQSPSTLPTQVLRAPPVRIAPPVTIRQ

VVPVFAAPPVRKEDIPSVRNEDVPTVIASALPNKSPAQVEEAASTIAKNLREIE

TVQSIEQLKI*

>DRB2_Vitis_vinifera[GSVIVG01025192001]

MYKNQLQELAQRSCFNLPSYTCIREGPDHAPRFKATVNFNGEIFESPN

YCTTLRQAEHSAAEVALNSLSNRGPSHSLAARILDETGVYKNLLQEIAQRVG

APLPQYTTFRSGLGHLPVFTGTVELAGITFTGEPAKNKKQAEKNAAMAAWSS

LKQLAKEAANSSSETENNDELEQITIARALLNYRLKEKMAIANSPSGTLSFPK

KFPIQNSRLFSPQLPPVATSKILPLICQKTPPRNRPPSPTANDGSMPSSQPRSRPT

PPPEIPCSRGSSLCSYPAV*

>DRB2_Zea_mays[GRMZM2G027462]

MYKNQLQELAQRSCFNLPSYTCLREGPDHAPRFKAAVNFNGEQFESP

GFFSTLRQAEHAAAEVALAALARRGPSYSLAARILDETGVYKNLLQEVAQRV

GAPLPLYTTERSGVGHLPVFTCTVELAGITFTGDHAKNKKQAEKNAASAAWS

ALKQLVREEVNSSNETENSDEQEQIRIARALLNYRLKEKMAMANYPHVSPFP

KKFPMQPERKPSFGQSSQSSYSKILPLFRPKSNSRSRPESPASTDGASQMPGRA

MDIHTPSPRSRFPAAEAAPYVPVGHFRMPCHSMAPSVTIRTAVPVFSAPPLPPP

SARPQQLPPLMSHPPPIRMASPVRMRPASPMFASSAPAQGPKLVRPVQLKDV

QDQPRKETAPSVIPVQVKDVQYQSRKSSMSPVIPVVVKDAQRQPLPVQTKDV

QTQTQAPKESLADPIPAIRPAVKIDLPAQGKKEASAGATSEAPSSATGNNGAM

ERGTSSDVLLARRSRAAVDGDGDKVEAKHEAEAQAVAEAAIRQLEIN*

Supplementary Information 3 Sequence of synthesized DNA used to construct vectors to transform *drb1* mutants.

Each sequence is identified as a "gBlock" (Integrated DNA Technologies, IDT) of the DRB gene (DRBgBlock), and is followed by three identifiers that specify its components. An example is DRBgBlock-1, the structure of which is 1.2-1.L-1.1, meaning it contains a continuous sequence (5' to 3') corresponding to the RDB**1** of DRB**2** followed by the inter-domain **l**oop of DRB**1** and then RDB**1** of DRB**1**.

DRBgBlock-1 1.2-1.L-1.1

atCAATTGctatTTATGCAAGAACCTACTTCAAGAATACGCTCAAAAGATGAA
TTACGCGATTCCATTGTATCAGTGCCAGAAGGTCGAAACTCTTGGGAGAG
TTACACAATTCACATGTACTGTAGAGATTGGAGGCATAAAGTACACAGGA
GCTGCAACAAGAACTAAAAAAGATGCTGAGATTAGCGCTGGGAGAACTG
CTCTTTTAGCGATCCAGTCATCCAGTGAGCTAAGCCAATGTGTTTCACAAC
CTGTTCACGAAACGGGAGTTTTCAAAAGTCGGTTGCAGGAGTATGCTCAG
AAGTACAAGCTCCCAACGCCTGTTTATGAGATCGTTAAAGAAGGCCCTTC
ACACAAATCTTTATTTCAATCGACTGTGATACTGGATGGTGTCAGATATAA
TTCTTTGCCTGGATTCTTCAATCGTAAGGCTGCAGAGCAATCAGCTGCCGA
GGTTGCTCTCCGGGAATTAGCAAAAACTAGTtaatgaGGATCCat

DRBgBlock-4 2.1-1.L-1.2

atCAATTGctatATGTATAAGAACCAGCTACAAGAGTTGGCTCAGAGGAGCTG
CTTTAATCTTCCTTCGTATACTTGTATTAGGGAAGGTCCTGACCACGCGCC
GCGATTCAAGGCTACTGTTAACTTTAACGGCGAGATCTTTGAGAGTCCTCA
GTATTGTTCTACTCTTCGTCAAGCTGAACACTCTGCTGCTGAAGTTGCTCT

CAATGCTCTCTCTAATTCCAGTGAGCTAAGCCAATGTGTTTCACAACCTGT

TCACGAAACGGGATTATGCAAGAACCTACTTCAAGAATACGCTCAAAAGA

TGAATTACGCGATTCCATTGTATCAGTGCCAGAAGGTCGAAACTCTTGGG

AGAGTTACACAATTCACATGTACTGTAGAGATTGGAGGCATAAAGTACAC

AGGAGCTGCAACAAGAACTAAAAAAGATGCTGAGATTAGCGCTGGGAGA

ACTGCTCTTTTAGCGATCCAGTCAACTAGTtaatgaGGATCCat

DRBgBlock-5 1.1-1.L-2.2

atatCAATTGctatGTTTTCAAAAGTCGGTTGCAGGAGTATGCTCAGAAGTACA

AGCTCCCAACGCCTGTTTATGAGATCGTTAAAGAAGGCCCTTCACACAAA

TCTTTATTTCAATCGACTGTGATACTGGATGGTGTCAGATATAATTCTTTG

CCTGGATTCTTCAATCGTAAGGCTGCAGAGCAATCAGCTGCCGAGGTTGC

TCTCCGGGAATTAGCAAAATCCAGTGAGCTAAGCCAATGTGTTTCACAAC

CTGTTCACGAAACGGGAGTGTATAAGAATCTTTTGCAAGAGATAGCTCAA

AGAGTGGGAGCTCCTTTACCGCGATATACTACTTTCAGGTCAGGTCTTGGT

CACCAACCTGTGTTTACTGGTACTGTAGAATTGGCTGGAATTACGTTCACT

GGAGATCCAGCTAAGAACAAGAAGCAAGCAGAGAAGAATGCTGCAATGG

CTGCTTGGTCTTCCCTAAAACAAACTAGTtaatgaGGATCCata

DRBgBlock-6 2.2-1.L-1.2

atatCAATTGctatGTGTATAAGAATCTTTTGCAAGAGATAGCTCAAAGAGTGG

GAGCTCCTTTACCGCGATATACTACTTTCAGGTCAGGTCTTGGTCACCAAC

CTGTGTTTACTGGTACTGTAGAATTGGCTGGAATTACGTTCACTGGAGATC

CAGCTAAGAACAAGAAGCAAGCAGAGAAGAATGCTGCAATGGCTGCTTG

GTCTTCCCTAAAACAATCCAGTGAGCTAAGCCAATGTGTTTCACAACCTGT

TCACGAAACGGGATTATGCAAGAACCTACTTCAAGAATACGCTCAAAAGA

TGAATTACGCGATTCCATTGTATCAGTGCCAGAAGGTCGAAACTCTTGGG

AGAGTTACACAATTCACATGTACTGTAGAGATTGGAGGCATAAAGTACAC

AGGAGCTGCAACAAGAACTAAAAAAGATGCTGAGATTAGCGCTGGGAGA

ACTGCTCTTTTAGCGATCCAGTCAACTAGTtaatgaGGATCCata

DRBgBlock-7 4.1-1.L-1.2

atCAATTGctatGTATACAAAGGTCAACTGCAAGCGTATGCCCTGCAACATAA

TCTGGAGCTACCAGTGTATGCGAATGAGAGAGAAGGGCCTCCTCATGCTC

CTAGATTTAGATGTAATGTTACATTCTGTGGACAGACTTTCCAGAGCTCTG

AATTCTTTCCGACACTAAAATCGGCTGAACATGCCGCTGCAAAAATTGCA

GTTGCTTCTTTGACGCCATCCAGTGAGCTAAGCCAATGTGTTTCACAACCT

GTTCACGAAACGGGATTATGCAAGAACCTACTTCAAGAATACGCTCAAAA

GATGAATTACGCGATTCCATTGTATCAGTGCCAGAAGGTCGAAACTCTTG

GGAGAGTTACACAATTCACATGTACTGTAGAGATTGGAGGCATAAAGTAC

ACAGGAGCTGCAACAAGAACTAAAAAAGATGCTGAGATTAGCGCTGGGA

GAACTGCTCTTTTAGCGATCCAGTCAACTAGTtaatgaGGATCCat

DRBgBlock-8 1.1-1.L-4.2

atatCAATTGctatGTTTTCAAAAGTCGGTTGCAGGAGTATGCTCAGAAGTACA

AGCTCCCAACGCCTGTTTATGAGATCGTTAAAGAAGGCCCTTCACACAAA

TCTTTATTTCAATCGACTGTGATACTGGATGGTGTCAGATATAATTCTTTG

CCTGGATTCTTCAATCGTAAGGCTGCAGAGCAATCAGCTGCCGAGGTTGC

TCTCCGGGAATTAGCAAAATCCAGTGAGCTAAGCCAATGTGTTTCACAAC

CTGTTCACGAAACGGGAGCCTACAAGAACCTGTTACAAGAAATTGCACAG

AAAGAGAGTTCTCTGTTACCATTTTATGCAACTGCTACATCTGGTCCATCG
CATGCGCCTACTTTTACTTCAACTGTTGAGTTTGCTGGTAAAGTTTTCAGT
GGAGAAGAGGCGAAAACCAAAAAGTTGGCTGAAATGAGCGCTGCTAAAG
TTGCATTCATGAGTATCAAAAATACTAGTtaatgaGGATCCata

DRBgBlock-9 4.2-1.L-1.2

atatCAATTGctatGCCTACAAGAACCTGTTACAAGAAATTGCACAGAAAGAG
AGTTCTCTGTTACCATTTTATGCAACTGCTACATCTGGTCCATCGCATGCG
CCTACTTTTACTTCAACTGTTGAGTTTGCTGGTAAAGTTTTCAGTGGAGAA
GAGGCGAAAACCAAAAAGTTGGCTGAAATGAGCGCTGCTAAAGTTGCATT
CATGAGTATCAAAAATTCCAGTGAGCTAAGCCAATGTGTTTCACAACCTG
TTCACGAAACGGGATTATGCAAGAACCTACTTCAAGAATACGCTCAAAAG
ATGAATTACGCGATTCCATTGTATCAGTGCCAGAAGGTCGAAACTCTTGG
GAGAGTTACACAATTCACATGTACTGTAGAGATTGGAGGCATAAAGTACA
CAGGAGCTGCAACAAGAACTAAAAAAGATGCTGAGATTAGCGCTGGGAG
AACTGCTCTTTTAGCGATCCAGTCAACTAGTtaatgaGGATCCata

DRBgBlock-10 1.1-2.L-1.2

atCAATTGctatGTTTTCAAAAGTCGGTTGCAGGAGTATGCTCAGAAGTACAA
GCTCCCAACGCCTGTTTATGAGATCGTTAAAGAAGGCCCTTCACACAAAT
CTTTATTTCAATCGACTGTGATACTGGATGGTGTCAGATATAATTCTTTGC
CTGGATTCTTCAATCGTAAGGCTGCAGAGCAATCAGCTGCCGAGGTTGCT
CTCCGGGAATTAGCAAAACGTGGTCCTTCTCACTCTCTTGCCGCCAGGATC
TTGGATGAGACGGGTTTATGCAAGAACCTACTTCAAGAATACGCTCAAAA
GATGAATTACGCGATTCCATTGTATCAGTGCCAGAAGGTCGAAACTCTTG

GGAGAGTTACACAATTCACATGTACTGTAGAGATTGGAGGCATAAAGTAC
ACAGGAGCTGCAACAAGAACTAAAAAAGATGCTGAGATTAGCGCTGGGA
GAACTGCTCTTTTAGCGATCCAGTCAACTAGTtaatgaGGATCCat

DRBgBlock-11 1.1-4.L-1.2

atataaCAATTGctatGTTTTCAAAAGTCGGTTGCAGGAGTATGCTCAGAAGTAC
AAGCTCCCAACGCCTGTTTATGAGATCGTTAAAGAAGGCCCTTCACACAA
ATCTTTATTTCAATCGACTGTGATACTGGATGGTGTCAGATATAATTCTTT
GCCTGGATTCTTCAATCGTAAGGCTGCAGAGCAATCAGCTGCCGAGGTTG
CTCTCCGGGAATTAGCAAAACAAAGTCCAGAGGGAATTGATGTTTTATGC
AAGAACCTACTTCAAGAATACGCTCAAAAGATGAATTACGCGATTCCATT
GTATCAGTGCCAGAAGGTCGAAACTCTTGGGAGAGTTACACAATTCACAT
GTACTGTAGAGATTGGAGGCATAAAGTACACAGGAGCTGCAACAAGAAC
TAAAAAAGATGCTGAGATTAGCGCTGGGAGAACTGCTCTTTTAGCGATCC
AGTCAACTAGTtaatgaGGATCCatataa

DRBgBlock-12 4.1-1.L-4.2

atatCAATTGctatGTATACAAAGGTCAACTGCAAGCGTATGCCCTGCAACATA
ATCTGGAGCTACCAGTGTATGCGAATGAGAGAGAAGGGCCTCCTCATGCT
CCTAGATTTAGATGTAATGTTACATTCTGTGGACAGACTTTCCAGAGCTCT
GAATTCTTTCCGACACTAAAATCGGCTGAACATGCCGCTGCAAAAATTGC
AGTTGCTTCTTTGACGCCATCCAGTGAGCTAAGCCAATGTGTTTCACAACC
TGTTCACGAAACGGGAGCCTACAAGAACCTGTTACAAGAAATTGCACAGA
AAGAGAGTTCTCTGTTACCATTTTATGCAACTGCTACATCTGGTCCATCGC
ATGCGCCTACTTTTACTTCAACTGTTGAGTTTGCTGGTAAAGTTTTCAGTG

GAGAAGAGGCGAAAACCAAAAAGTTGGCTGAAATGAGCGCTGCTAAAGT

TGCATTCATGAGTATCAAAAATACTAGTtaatgaGGATCCata

Supplementary Information 4 List of oligos used in this work.

Oligo name	Specificity*	Sequence (5' to 3')
ACT2_qPCR-FW	AT3G18780	GCAGATGTGGATCTCCAAGGCCGA
ACT2_qPCR-RV	AT3G18780	TTTCTGTGAACGATTCCTGGACCTGCC
DRB1_qPCR-FW	AT1G09700	TCCACTGATGTTTCCTCTGG
DRB1_qPCR-RV	AT1G09700	GATCTCATAAACAGGCGTTGG
Chimera3'UTR_qPCR-FW	Chimera 3'UTR	ATAATTCACTGGCCGTCGTTTTAC
Chimera3'UTR_qPCR-RV	Chimera 3'UTR	GAGCTCGTTTTCCCAGTCACG
ARPN_qPCR-FW	AT2G02850	ATGGCCAAGGGAAGAGGCAGT
ARPN_qPCR-RV	AT2G02850	ACGTCGCCGGCTCTAAAGTGTTTG
ATHB14_qPCR-FW	AT2G34710	AGAACGTTCCACCCGCTGTGCT
ATHB14_qPCR-RV	AT2G34710	CAGCAGCATAAGCATCCACGCCA
CSD2_qPCR-FW	AT2G28190	CAATGCCGATGGCGTGGCAGAA
CSD2_qPCR-RV	AT2G28190	GCCACCCTTTCCGAGGTCATCCTT
REV_qPCR-FW	AT5G60690	TGCACCAACGACTCTGGCTCCT
REV_qPCR-RV	AT5G60690	AGCAGCATTAGGCCCAGCTCCA
SL_RT-snoR101	SnoR101	GTCGTATCCAGTGCAGGGTCCGAGGTATTC GCACTGGATACGACAGCATC
SL_RT-miR164abc	miR164abc	GTCGTATCCAGTGCAGGGTCCGAGGTATTC GCACTGGATACGACYGCACG
SL_RT-miR165/166	miR165/166	GTCGTATCCAGTGCAGGGTCCGAGGTATTC GCACTGGATACGACGGGGRA
SL_RT-miR398a-c	miR398a-c	GTCGTATCCAGTGCAGGGTCCGAGGTATTC GCACTGGATACGACMAGGGG
SL_RT-miR408	miR408	GTCGTATCCAGTGCAGGGTCCGAGGTATTC GCACTGGATACGACGCCAGG
URP	SL adaptor	CCAGTGCAGGGTCCGAGGT
SL_qPCR-snoR101	SnoR101	CACAGGTAAGTTCGCTTGTTG
SL_qPCR-miR164ab	miR164ab	TGGAGAAGCAGGGCACGTGC

SL_qPCR-miR165ab/166a-g	miR165ab/166a-g	TCGGACCAGGCTTCATYCCC
SL_qPCR-miR398bc	miR398bc	GGTGTGTTCTCAGGTCACCCCTG
SL_qPCR-miR408	miR408	ATGCACTGCCTCTTCCCTGGC
drb1_gen-FW	SALK_064863	CTTCTTGGAAATTGGATTGCAGTG
drb1_gen-RV	SALK_064863	GCCCCCTAACGTATTCTCACAGC
PATcassetteF	35S promoter	atctaaCGGTCCGaacgtggatacttggcagtgg
PATcassetteR	NOS terminator	caggatGATATCCGGACCGgatctagtaacatagatgacaccgc
DRB1gateF	DRB1/AT1G09700	CACCATGACCTCCACTGATGTTTCC
DRB1dbd2R	DRB1/AT1G09700	GCTAGCTGACTGGATCGCTAAAAGAG
DRB1ctR	DRB1/AT1G09700	TGCGTGGCTTGCTTCTGTC
DRB2gateF	DRB2/AT2G28380	CACCATGTATAAGAACCAGCTAC
DRB2ctR	DRB2/AT2G28380	GATCTTTAGGTTCTCCAGTC
DRB1proFW	upstream DRB1	aagttgGCATGCaGAACGAAAAGGAAGGTGG
DRB1proRV	DRB1/AT1G09700	ttatatGTCGACaCAATTGGAAACACCTGAGCAGG
pENL42R1-F	pEN::L4-2-R1	ggcggccgcactagtgatatc
pENL42R1-R	pEN::L4-2-R1	tagtagACTAGTtatagtGTCGACtggtaaCCGCGGtgacgtCTCGAGtgatatGCATGCtatagtgtcacctaaatc

* Refers to locus amplified or DNA probe for northern blot.

ABRC stock number is provided for genotyping oligos.
